# Changes in the expression of splicing factor transcripts and variations in alternative splicing are associated with lifespan in mice and humans

**DOI:** 10.1111/acel.12499

**Published:** 2016-06-30

**Authors:** Benjamin P. Lee, Luke C. Pilling, Florence Emond, Kevin Flurkey, David E. Harrison, Rong Yuan, Luanne L. Peters, George A. Kuchel, Luigi Ferrucci, David Melzer, Lorna W. Harries

**Affiliations:** ^1^ RNA‐Mediated Mechanisms of Disease; ^2^ Epidemiology and Public HealthInstitute of Biomedical and Clinical SciencesUniversity of Exeter Medical SchoolUniversity of ExeterDevonUK; ^3^ The Jackson Laboratory Nathan Shock Centre of Excellence in the Basic Biology of AgingBar HarborMEUSA; ^4^ UConn Centre on AgingUniversity of Connecticut Health CentreFarmingtonCTUSA; ^5^ National Institute on AgingBaltimoreMDUSA; ^6^ Present address: Geriatric Research DivisionDepartment of Internal MedicineSouthern Illinois University School of MedicineSpringfieldILUSA

**Keywords:** isoforms, lifespan, longevity, mouse, mRNA splicing, splicing factors

## Abstract

Dysregulation of splicing factor expression and altered alternative splicing are associated with aging in humans and other species, and also with replicative senescence in cultured cells. Here, we assess whether expression changes of key splicing regulator genes and consequent effects on alternative splicing are also associated with strain longevity in old and young mice, across 6 different mouse strains with varying lifespan (A/J, NOD.B10Sn‐H2^b^/J, PWD.Phj, 129S1/SvlmJ, C57BL/6J and WSB/EiJ). Splicing factor expression and changes to alternative splicing were associated with strain lifespan in spleen and to a lesser extent in muscle. These changes mainly involved hnRNP splicing inhibitor transcripts with most changes more marked in spleens of young animals from long‐lived strains. Changes in spleen isoform expression were suggestive of reduced cellular senescence and retained cellular proliferative capacity in long‐lived strains. Changes in muscle isoform expression were consistent with reduced pro‐inflammatory signalling in longer‐lived strains. Two splicing regulators, *HNRNPA1* and *HNRNPA2B1,* were also associated with parental longevity in humans, in the InCHIANTI aging study. Splicing factors may represent a driver, mediator or early marker of lifespan in mouse, as expression differences were present in the young animals of long‐lived strains. Changes to alternative splicing patterns of key senescence genes in spleen and key remodelling genes in muscle suggest that correct regulation of alternative splicing may enhance lifespan in mice. Expression of some splicing factors in humans was also associated with parental longevity, suggesting that splicing regulation may also influence lifespan in humans.

## Introduction

Aging is a dynamic, multisystem process, which is highly heterogeneous in humans with some people surviving disease‐free until advanced age whilst others succumb to age‐related conditions in mid‐life. The factors underlying individual lifespan are currently unclear, but increasing our understanding of determinants of longevity and ‘healthspan’ are key aims for the future.

Correct expression and regulation of genes is critical for maintenance of cellular and organismal function. Alternative splicing, the process by which single genes can make multiple gene products in an adaptive and reactive fashion is a key part of this process (Cartegni *et al*., 2002). Indeed, breakdown in the regulation of mRNA splicing is a prominent feature in many age‐related diseases such as Alzheimer's disease, Parkinson's disease and several tumour types (Scuderi *et al*., 2014; Danan‐Gotthold *et al*., 2015; Lisowiec *et al*., 2015; Lu *et al*., 2015). This may indicate that defects in the splicing machinery may cause the cellular response to stress to be less specific, with effects on cellular resiliency and accumulation of DNA damage. We have previously identified deregulation of splicing factor expression and alternative splicing as a key factor in normal human and cellular aging (Harries *et al*., 2011; Holly *et al*., 2013). Alternatively expressed isoforms also demonstrate tissue‐specific differences in aging, as they do for many other phenomena (Holly *et al*., 2013). Splicing factors themselves demonstrate high species conservation (Barbosa‐Morais *et al*., 2006), whereas patterns of alternative splicing are partially determined by genetic differences and may be species specific. Splicing patterns show drastically more interspecies variability than gene expression with only 50% of alternatively expressed isoforms being conserved between species (Barbosa‐Morais *et al*., 2012). Alternatively regulated splice sites demonstrating temporal, spatial or reactive expression are less likely to show species conservation (Garg & Green, 2007).

Several splicing factors have been suggested to be involved in organismal lifespan. The pre‐mRNA processing factor 19 homologue (SNEV) protein, important for spliceosome assembly and mRNA processing, has been shown to suppress cellular senescence and suppress apoptosis when phosphorylated by the ataxia‐telangiectasia (ATM) kinase in endothelial cells (Dellago *et al*., 2012). The DNA damage protein ATM also appears to be an important regulator of splicing factor expression in our previous work, since targeted gene knockdown of the *ATM* gene resulted in increased levels of splicing factor expression in fibroblasts (Holly *et al*., 2013). There is also evidence from systemic models. A network‐based model of genes altered by calorific restriction across 17 tissues in mice revealed that the largest and most responsive gene regulatory module was associated with mRNA processing, with a disproportionally large number of genes being involved in splicing, metabolism, processing and biosynthesis of mRNA (Swindell, 2009). Finally, a study of the relationship between copy number variation (CNV) and longevity in humans revealed that lifespan‐associated CNVs were preferentially located in or near genes encoding proteins involved in splicing control. This led them to conclude that genetic variation that disrupts the processes of alternative splicing may have long‐term effects on lifespan (Glessner *et al*., 2013).

The study of inbred strains of mice has proven fruitful in uncovering factors associated with lifespan, as for other phenotypes. The Jackson Laboratory has characterized 30 strains of mice, for aging and longevity‐related traits (Yuan *et al*., 2009, 2011). Initial work with this resource identified that plasma IGF1 levels were related to lifespan in rodents (Yuan *et al*., 2009). This collection, together with the associated repository of mouse phenome data, represents a rich resource (Bogue *et al*., 2014). Here, we have harnessed this resource to assess the contribution of regulation of alternative splicing to longevity in mice.

**Figure 1:**
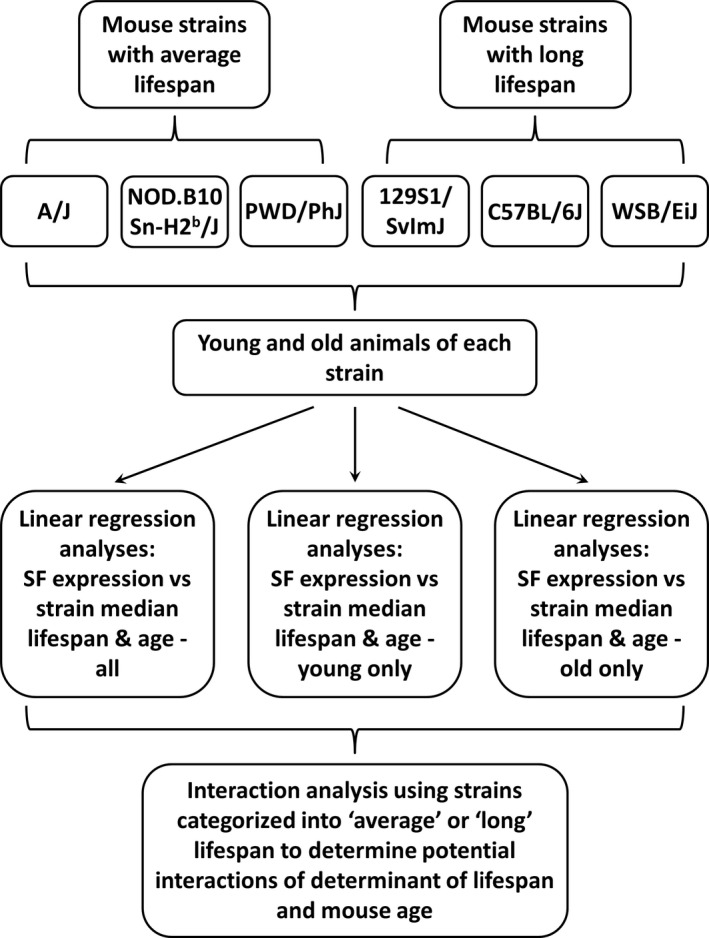
Schematic of study design. This figure shows the experimental strategy employed to assess the effects of strain longevity and mouse age on the expression of an *a priori* panel of splicing factors (SFs).

We have systematically assessed the expression of splicing factors previously demonstrated to be altered in human aging in relation to lifespan in six mouse strains of different longevities. Our study design is illustrated in Fig. 1. Splicing factor expression and alternative splicing of key genes are associated with lifespan in mouse spleen tissue and to a lesser extent in mouse muscle, suggesting that aging effects may be driven by immune tissues. Some strain differences in expression are most marked in the young mice, suggesting that they may represent determinants or early markers of longevity rather than representing secondary effects of aging. We also identified differences in expression levels of alternatively expressed isoforms of key aging genes indicative of reduced cellular senescence, maintained cellular proliferative capacity (spleen) and reduced pro‐inflammatory signalling (muscle) in long‐lived mouse strains. Two splicing factors, *HNRNPA2B1* and *HNRNPA1,* were also associated with parental longevity in a large population study of aging, suggesting that regulation of splicing may also be involved in lifespan in human populations.

## Results

### Splicing factor transcript expression is associated with lifespan in mouse spleen and to a lesser extent in muscle tissues

We assessed splicing factors that we have previously demonstrated to be altered in human aging in relation to lifespan in 6 mouse strains of different longevities and in samples from both old and young mice as in Fig. 1. The expression of 8/15 and 3/15 splicing factors was associated with strain lifespan in mouse spleen and muscle tissue, respectively.

**Figure 2:**
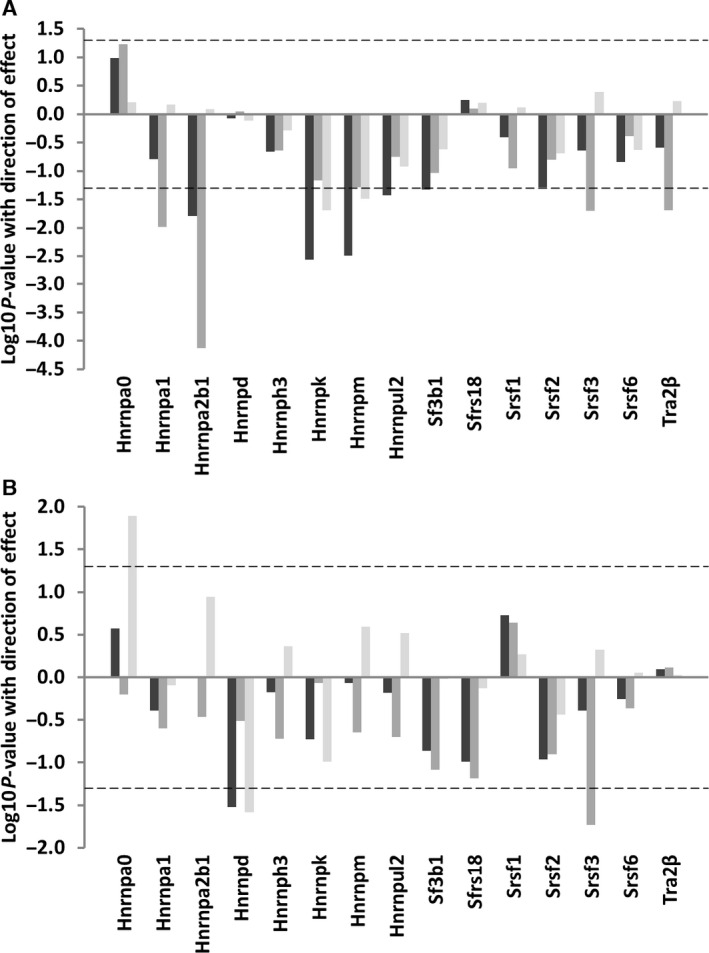
Splicing factor expression according to mouse lifespan. This plot illustrates association between median lifespan and slicing factor expression in total RNA from spleen (A) or muscle (B) tissues in mice of 6 strains of different longevities, as assessed by linear regression against median strain lifespan. The identity of specific splicing factors is given on the *x*‐axis. The log10 *P*‐values for associations between lifespan and splicing factor expression from mice of strains with different lifespans and of different ages are given on the *y*‐axis. Direction of effect is also indicated; data appearing above the zero line on the *y*‐axis represent positive associations, whilst data appearing below the zero line represent negative associations. Analysis including all animals in the sample is given by dark grey bars, in young animals only by medium grey bars and in old animals only by light grey bars. The dotted line refers to a *P*‐value cut‐off for statistical significance of *P* = 0.05.

In spleen, we found associations between strain lifespan and the expression of the *Hnrnpa1, Hnrnpa2b1, Hnrnpk, Hnrnpm, Hnrnpul2, Sf3b1, Srsf3* and *Tra2β* genes (beta coefficients −0.40, −0.26, −0.32, −0.31, −0.22, −0.13 and −0.36; *P* = 0.01, 0.02, 0.003, 0.003, 0.04, 0.05, 0.02 and 0.02, respectively; Fig. 2A, Table S1). When an analysis was carried out to assess interactions between strain lifespan and mouse age, we found effects in both young and old mice of long‐lived strains for all associated genes except *Hnrnpul2*. In the case of *Hnrnpa1* and *Hnrnpa2b1* genes, these differences were more marked in the young mice of long‐lived strains (beta coefficients −0.14 and −0.19, *P* = 0.001 and < 0.0001 in the young long‐lived mice compared with −0.09 and −0.12; *P* = 0.01 and 0.02 for the old long‐lived mice for *Hnrnpa1* and *Hnrnpa2b1,* respectively; Table S9). For *Srsf3*, the associations between splicing factor expression/age and splicing factor expression/strain median lifespan appear to be comparable, whereas for *Tra2β*, our data suggest that the effects of age are stronger than those of lifespan (Table S9). The majority (5/8) of the splicing factors demonstrating association of expression differences with lifespan belonged to the hnRNP class of splicing inhibitors, with only 2 splicing activators (*Srsf3* and *Tra2β*) showing expression changes with lifespan. The remaining associated splicing factor, *Sf3b1,* encodes a component of the U2 snRNP in the core spliceosome complex, rather than a splicing regulator. When data were assessed by binary logistic regression to allow for nonlinearity of response, all but one (*Hnrnpul2*) of the splicing regulators associated with lifespan in spleen remained associated with median strain lifespan (Table S10).

Fewer splicing factors were associated with strain lifespan in muscle tissue (Fig. 2B, Table S2). *Hnrnpa0* expression was found to be positively correlated with long life (beta coefficient 0.36; *P* = 0.01), whereas *Hnrnpd* and *Srsf3* transcripts both demonstrated reduced expression (beta coefficients −0.24 and −0.40; *P* = 0.03 and 0.02, respectively). Interaction analysis revealed that the *Srsf3* effect was again driven by effects in the young animals of the long‐lived strains (beta coefficient −0.14, *P* = 0.01 in the young long‐lived mice compared with beta coefficient −0.05, *P* = 0.28 in the old long‐lived mice; Table S9). Again 2/3 lifespan‐associated splicing factors represented hnRNP splicing inhibitors rather than SRSF splicing activators. When data were assessed by binary logistic regression to allow for nonlinearity of response, all of the splicing regulators associated with lifespan in muscle remained associated with median strain lifespan (Table S11). Effects were tissue specific, with little overlap between lifespan‐associated splicing factors in spleen and those in muscle, with only *Srsf3* common to both data sets.

### Alternatively spliced genes demonstrate longevity‐associated isoform changes in mouse spleen and muscle tissue

**Figure 3:**
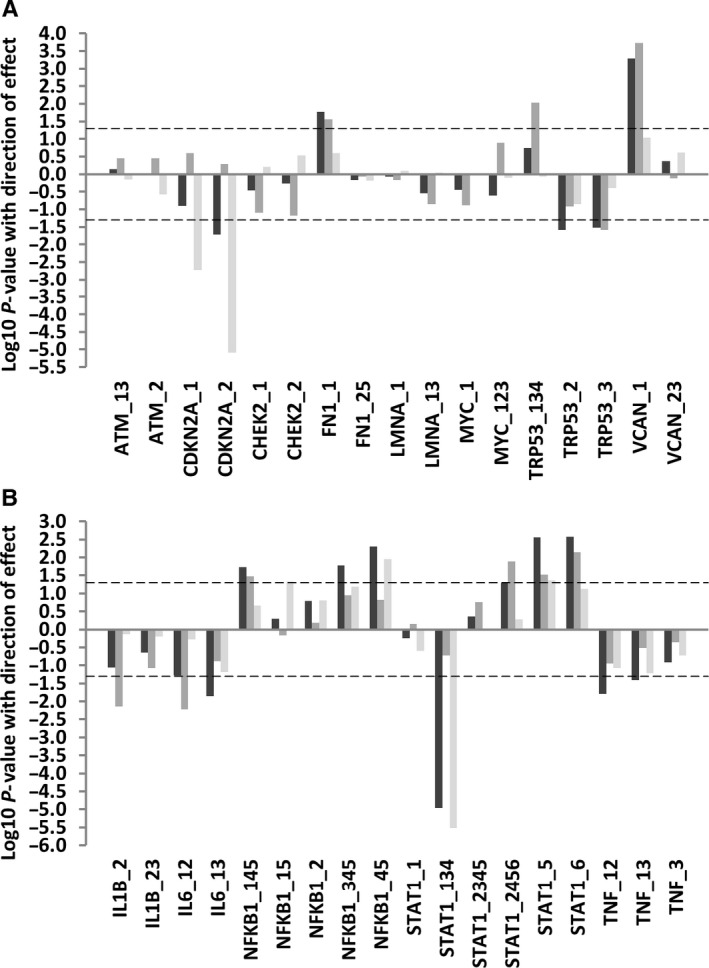
The expression of alternative isoforms of key genes according to mouse lifespan. This plot illustrates association between median lifespan and the expression of alternatively expressed isoforms of key genes in total RNA from spleen (A) or muscle (B) tissues in mice of 6 strains of different longevities as assessed by linear regression against median strain lifespan. The identity of specific splicing factors is given on the *x*‐axis. The log10 *P*‐values for associations between lifespan and splicing factor expression from mice of strains with different lifespans and of different ages are given on the *y*‐axis. Direction of effect is also indicated; data appearing above the zero line on the *y*‐axis represent positive associations, whilst data appearing below the zero line represent negative associations. Analysis including all animals in the sample is given by dark grey bars, in young animals only by medium grey bars and in old animals only by light grey bars. The dotted line refers to a *P*‐value cut‐off for statistical significance of *P* = 0.05.

In spleen, we found splicing differences in association with lifespan for 4/8 genes tested (Fig. 3A; Table S3). Both uc008toi.1 and uc008toh.1 transcripts encoding p16INK4A and p14ARF isoforms of the *Cdkn2a* gene were expressed at lower levels in the long‐lived strains (beta coefficients −0.43 and −0.59; *P* = 0.002 and < 0.0001 for uc008toi.1 [p16INK4A] and uc008toh.1 [p14ARF], respectively). Analysis of the interaction of strain longevity and mouse age revealed that although the effects on *Cdkn2a* isoform expression increased with age as expected in both average‐lived and long‐lived mice, the increase in expression was much less marked in the old long‐lived mice than in old mice of average lifespan (beta coefficients 0.44 and 0.50, *P* = <0.0001 and < 0.0001 for the old average‐lived mice compared with beta coefficients 0.22 and 0.27, *P* = 0.01 and 0.001 for the old long‐lived mice; Table S9).

We also found expression of the uc007bju.2 isoform only of the *Fn1* gene to be increased in the long‐lived strains (beta coefficient 0.25, *P* = 0.02). There is increased expression of the long isoform of the *Trp53* gene encoding full‐length p53 (uc007jql.2/uc007jqn.2), but reduced expression of the truncated *p53AS* isoforms (uc011xww.1/uc007jqm.2; beta coefficients 0.41 and −0.35, *P* = 0.009 and 0.03 for full‐length *Trp53* and truncated *p53AS* isoforms, respectively). Assessment of the interaction between strain longevity and mouse age revealed that expression of the full‐length p53 isoform is only significantly increased in the young animals of long‐lived strains (in comparison with young animals of short‐lived strains *P* = 0.004; the older animals were not significantly different to young short‐lived strains *P* > 0.05) and that diminished expression of p53AS was only significantly decreased in the old animals of long‐lived strains (*P* = 0.009, in comparison with young animals of short‐lived strains; other groups were *P* > 0.05, Table S9). Finally, expression of the full‐length uc007rjg.1 isoform of the *Vcan* gene is upregulated (beta coefficient 0.34, *P* = 0.001).

In mouse muscle, expression of isoforms of all five genes tested in relation to lifespan was altered (Fig. 3B, Table S4). First, expression of the full‐length uc008mht.1 isoform of the *Il1b* gene was reduced in the long‐lived strains (beta coefficient −0.46, *P* = 0.007), whilst the intron‐retained uc008mhu.1 *Il1b* isoform was unaffected. Interaction analysis revealed that this difference was limited to the young mice of the long‐lived strains (*P* = 0.004 for young long‐lived mice compared with *P* = 0.34 for the old long‐lived mice). Expression levels of the intron‐retained uc008wuv.1 and full‐length uc008wuw.1 isoforms of the *Il6* gene were also reduced (beta coefficient −0.48, *P* = 0.006 and −0.28, *P* = 0.01, respectively). Interaction analyses revealed that the effects on the intron‐retained isoform were significant in the young long‐lived mice only (*P* = 0.04 for young long‐lived mice compared with *P* = 0.72 for old long‐lived mice; Table S9).

Expression of the uc012cyg.1/uc008rlx.1 isoforms which encode the long full‐length forms of the *Nfkb1* gene was greater in long‐lived strains (beta coefficient 0.31, *P* = 0.005). No difference was seen in the expression of the noncoding truncated uc012cyf.1 *Nfkb1* isoform. Isoform usage for the *Stat1* gene in long‐lived strains differed; the uc007axz.1 and uc007aya.2 isoforms of *Stat1* that both encode ‘variant 2’ of the STAT1 protein demonstrated diminished expression in the long‐lived strains (beta coefficients −0.63, *P* = <0.0001), whereas the *Stat1* uc007ayc.2 isoform encoding ‘variant 1’ demonstrated increased expression in the long‐lived strains (beta coefficient 0.46, *P* = 0.007). Interaction analysis revealed that the decrease in *Stat1* variant 2 expression was present only in the old long‐lived mice (*P* = 0.01 in old long‐lived mice compared with *P* = 0.28 in the young long‐lived mice; Table S9). The greater *Stat1* variant 1 expression was driven by effects in the young long‐lived mice (*P* = 0.03 in young long‐lived mice compared with *P* = 0.95 in old long‐lived mice). Transcript uc007ayb.2, encoding *Stat1* ‘variant 3’, was also upregulated (beta coefficient 0.33, *P* = 0.005) although this was seen in both young and old animals of long‐lived strains. Finally, expression of both the uc012arb.2 and uc008cgs.2 isoforms of the *Tnf* gene which encode TNF variants 1 and 2 were reduced in muscle (beta coefficients −0.27 and −0.18, *P* = 0.02 and 0.04 for uc012arb.2 and uc008cgs.2, respectively). Interaction analyses revealed that the effect for *Tnf* variant 1 is most marked in the old long‐lived animals (*P* = 0.008 in the old long‐lived animals compared with *P* = 0.05 in the young long‐lived animals, Table S9).

### Few splicing factors are associated with age in mouse spleen and muscle tissues

**Figure 4:**
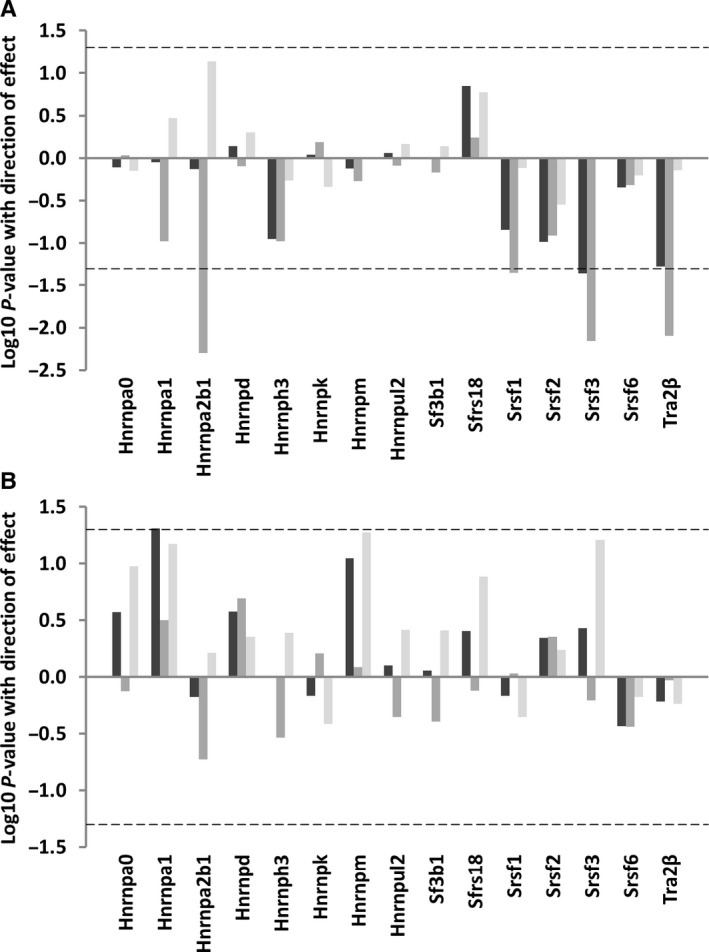
Splicing factor expression according to mouse age. This plot illustrates association between age and splicing factor expression in total RNA from spleen (A) or muscle (B) tissues in young (6 months) vs. old (20–22 months) mice. The identity of specific splicing factors is given on the *x*‐axis. The log10 *P*‐values for associations between age and splicing factor expression from mice of different ages and of different strains are given on the *y*‐axis. Direction of effect is also indicated; data appearing above the zero line on the *y*‐axis represent positive associations, whilst data appearing below the zero line represent negative associations. Analysis including all animals in the sample is given by dark grey bars, in animals of average‐lived strains only by medium grey bars and in long‐lived animals only by light grey bars. The dotted line refers to a *P*‐value cut‐off for statistical significance of *P* = 0.05.

Associations between mouse age and splicing factor expression were less marked than those seen for strain longevity (Fig. 4A,B, Tables S5 and S6). In spleen, we found reduced expression of the *Hnrnpa2b1, Srsf1, Srsf3* and *Tra2β* transcripts in old mice (beta coefficients −0.46, −0.34, −0.45 and −0.53; *P* = 0.005, 0.05, 0.007 and 0.008, respectively). Effects on *Srsf1*,* Srsf3* and Tra2*β* expression were evident in old animals of both average‐lived and long‐lived strains, but interaction analysis revealed that the age‐associated difference in *Hnrnpa2b1* expression was most marked in the old animals of strains of average lifespan (beta coefficients −0.11, *P* = 0.02 in the old long‐lived animals compared to beta coefficient −0.13 *P* = <0.0001 in the old average‐lived animals). Age‐associated changes to splicing factor expression were much less evident in muscle tissue, with only *Hnrnpa1* demonstrating increased expression (beta coefficient 0.22, *P* = 0.05). Analysis of cluster patterns for age revealed considerable inter‐ and intrastrain heterogeneity in splicing factor expression (Fig. S1).

### Alternatively expressed isoforms demonstrate differential expression with age in mouse spleen and muscle tissue

**Figure 5:**
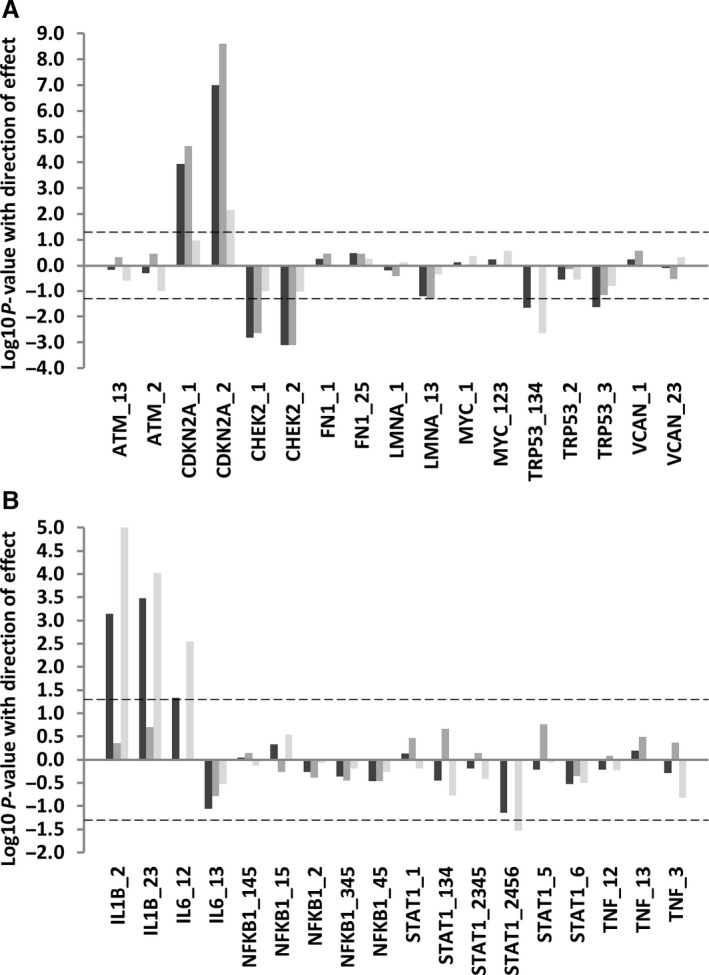
The expression of alternative isoforms of key genes according to mouse age. This plot illustrates association between the expression of age and the expression of alternatively expressed isoforms of key genes in total RNA from spleen (A) or muscle (B) tissues in young (6 months) vs. old (20–22 months) mice. The identity of isoforms is given on the *x*‐axis. The log10 *P*‐values for associations between age and the expression of alternative isoforms from mice of different ages and of different strains are given on the *y*‐axis. Direction of effect is also indicated; data appearing above the zero line on the *y*‐axis represent positive associations, whilst data appearing below the zero line represent negative associations. Analysis including all animals in the sample is given by dark grey bars, in animals of average‐lived strains only by medium grey bars and in long‐lived animals only by light grey bars. The dotted line refers to a *P*‐value cut‐off for statistical significance of *P* = 0.05.

Despite the small numbers of splicing factors demonstrating age‐associated differences in splicing factor expression, we noted differences in alternative splicing in both spleen and muscle from aged mice (Fig. 5A,B, Tables S7 and S8).

In mouse spleen, we identified increased expression of both uc008toi.1 and uc008toh.1 transcripts encoding p16INK4A and p14ARF isoforms of the *Cdkn2a* gene in the old animals as expected (beta coefficients 0.40 and 0.53, *P* = <0.0001 and 0 < 0.0001, respectively). Interaction analyses revealed the age‐associated increase in both *Cdkn2a* isoforms to be significantly reduced in old animals of long‐lived strains as described above. We also identified decreased expression of both uc008yrw.1 (full length) and uc008yrx.1 (exon skipped) isoforms of the *Chek2* gene with increasing age (beta coefficients −0.33 and −0.35, *P* = 0.02 and 0.01, respectively). Interaction analyses between mouse age and strain longevity revealed that reduced expression of the full‐length *Chek2* isoform was more marked in the old animals of the long‐lived strains (beta coefficient −0.26, *P* = <0.0001 in the old long‐lived animals compared with beta coefficient −0.24, *P* = 0.003 in the old average‐lived animals; Table S9). *Trp53* isoforms also demonstrated effects with age in mouse spleen. With age, there was a reduction in levels of transcripts encoding both full‐length p53 (uc007jql.2/ uc007jqn.2) and also those encoding the truncated alternatively spliced p53AS (uc007jqm.2) isoform (beta coefficients −0.24 and −0.24, *P* = 0.02 and 0.03, respectively).

In mouse muscle, we found increased expression of both the full‐length (uc008mht.1) and the intron‐inclusion (uc008mhu.1) *Il1b* transcripts with age (beta coefficients 0.37 and 0.39, *P* = 0.001 and < 0.0001). The old animals also demonstrated elevated expression of the uc008wuv.1 isoform of the *Il6* gene, which contains a retention of intron 4 relative to the consensus transcript (beta coefficient 0.44, *P* = 0.003). Old animals also demonstrated reduction of uc007ayd.2, uc007aya.2, uc007ayb.2 and uc007ayc.2 isoforms of the *Stat1* gene, with the effects on uc007aya.2 being revealed by interaction analysis between mouse age and strain lifespan to be present exclusively in the old animals of long‐lived strains (beta coefficient −0.17, *P* = 0.01; Table S9).

### The expression of some splicing factors is associated with parental longevity in a large human population

We examined the expression of 15 splicing factors identified through the mouse work described in this study and previous analyses (Holly *et al*., 2013) and determined that 2 genes, *HNRNPA2B1* and *HNRNPA1*, demonstrated associations of their expression with parental longevity, as defined in Dutta *et al*. (2013a) in the human InCHIANTI population study as well as in mice (Table 1). *HNRNPA2B1* transcripts demonstrated increased expression in the offspring of long‐lived parents (beta coefficients 0.12, *P* = 0.017), whereas *HNRNPA1* demonstrated reduced expression in the offspring of long‐lived parents (beta coefficient −0.09; *P* = 0.035, respectively; Table 1). *Hnrnpa1* demonstrates reduced expression in association with longevity in both man and mouse, whereas *Hnrnpa2b1* shows elevated expression with longevity in people, but reduced expression with longevity in mice.

**Table 1:** Associations between splicing factor expression and parental longevity in humans (the InCHIANTI population)

Gene name	Probe Id	Beta coefficient	95% Confidence intervals		*P*‐value
***HNRNPA2B1***	ILMN_1886493	0.116	0.020	0.212	**0.017**
***HNRNPA1***	ILMN_1676091	−0.091	−0.176	−0.006	**0.035**
***HNRNPA2B1***	ILMN_2369682	0.088	0.002	0.175	**0.044**
*TRA2B*	ILMN_1742798	0.092	−0.001	0.186	0.051
*HNRNPA1*	ILMN_1661346	0.069	−0.004	0.143	0.065
*SRSF1*	ILMN_1795341	0.082	−0.008	0.171	0.073
*HNRNPD*	ILMN_2321451	0.088	−0.010	0.186	0.078
*HNRNPK*	ILMN_1701753	−0.066	−0.176	0.043	0.232
*HNRNPUL2*	ILMN_2072091	0.076	−0.053	0.205	0.246
*HNRNPK*	ILMN_2378048	0.060	−0.058	0.178	0.319
*SRSF18*	ILMN_2161357	0.068	−0.072	0.209	0.341
*HNRNPUL2*	ILMN_1810327	0.055	−0.066	0.176	0.374
*HNRNPA1*	ILMN_2220283	0.034	−0.051	0.119	0.432
*SRSF2*	ILMN_1696407	0.047	−0.074	0.167	0.446
*HNRNPD*	ILMN_1751368	−0.034	−0.137	0.068	0.511
*SRSF6*	ILMN_1697469	0.033	−0.077	0.143	0.552
*SRSF6*	ILMN_1754304	0.030	−0.078	0.138	0.585
*HNRNPA0*	ILMN_1753279	−0.035	−0.164	0.095	0.598
*HNRNPA1*	ILMN_1663447	−0.034	−0.168	0.101	0.624
*HNRNPA1*	ILMN_1720745	0.025	−0.080	0.131	0.637
*HNRNPM*	ILMN_2385173	−0.029	−0.161	0.103	0.668
*SRSF6*	ILMN_1805371	−0.026	−0.149	0.096	0.673
*SF3B1*	ILMN_1712347	−0.022	−0.151	0.107	0.738
*SF3B1*	ILMN_1705151	0.017	−0.091	0.125	0.754
*HNRNPM*	ILMN_1745385	−0.016	−0.119	0.086	0.756
*HNRNPA1*	ILMN_2175075	−0.012	−0.126	0.102	0.838
*SRSF3*	ILMN_2389582	0.004	−0.118	0.125	0.951

The relationship of parental longevity with expression of 15 unique splicing factors in 405 individuals by multivariate linear regression. Genes demonstrating significant associations at *P* = <0.05 are indicated in bold underlined text.

### Genetic variation within Hnrnpa2b1 and Hnrnpa1 that is discrepant between strains is unlikely to contribute to differences in gene expression

We have carried out a bioinformatic analysis of the potential for genetic variation that is discrepant between strains of mice to affect the regulation of the *Hnrnpa2b1* and *Hnrnpa1* genes. These genes were selected because their expression is associated with longevity in both mouse and man. The *Hnrnpa2b1* gene harbours 14 genetic variants that are discordant between strains. Two of these variants, rs51031918 and rs252413833, are associated with changes to the binding efficiencies of SRSF2 and SRSF5 splicing enhancers (see Table S12). These changes are, however, very subtle and may not adversely affect SRSF2 or SRSF5 binding. Similarly, two *Hnrnpa1* variants (rs32398879 and rs50030666) are discordant between the strains. One of these, rs50030666, lies in a cassette exon which is intronic in some *Hnrnpa1* isoforms, but coding in others (see Table S12). The coding change causes the substitution of glycine residue for a similarly sized serine residue with equivalent charge. No other predicted effects of genetic variation on transcription factor binding, RNA regulatory elements (A‐rich elements, C to U RNA editing sites or microRNA binding sites) were identified for any variant studied.

## Discussion

Splicing factor expression has been shown to be conclusively associated with chronological age in humans (Harries *et al*., 2011) and also with cellular senescence in multiple human primary cell lines in culture (Holly *et al*., 2013), indicating that these factors may be linked with cellular plasticity and adaptability during the aging process. Here, we have assessed the potential relationships between splicing factor expression and alternative splicing with strain longevity across 6 mouse strains of variable medium to long lifespans, in both young and old animals. We also assessed associations between splicing factor expression and parental longevity in the offspring of long‐lived parents in humans. We have found that over half of the splicing factors tested are associated with longevity in mouse spleen, and to a lesser extent in mouse muscle and that these changes are accompanied by alterations to the profile of selected alternatively expressed isoforms in both tissues. Two splicing factors, *HNRNPA1* and *HNRNPA2B1,* also showed evidence of an association with parental longevity in humans. These results, to our knowledge, represent the first link between the regulation of alternative splicing and inherited longevity traits in mammals.

In spleen, 7/8 splicing factors tested demonstrated associations with strain longevity in both young and old animals, with effects being predominant in the young animals of longer‐lived strains suggesting that these differences in splicing factor expression are not the end result of aging processes as such, but rather may represent fundamental differences in factor expression that drive or contribute to the aging process. It is possible that changes happening early on in life may set the scene for future longevity, which is an interesting concept given that several genes such as *Foxo1,* known to be associated with extended lifespan, are developmental genes (Lunetta *et al*., 2007). It is very difficult to predict what the consequence of these changes will be to the overall level or pattern of splicing in long‐lived mice or humans, since splice site choice at any given exon: intron junction is determined by the balance of activators and repressors, and that this balance is individually determined for each splice site in each gene (Cartegni *et al*., 2002). However, diminished splicing factor expression may be beneficial in younger animals, since both SRSF and hnRNP splicing factors are known to have oncogenic features (Gautrey *et al*., 2015; Goncalves & Jordan, 2015; Guo *et al*., 2015). Lower splicing factor expression in younger animals may thus protect against an earlier death from malignancy.

We also found clear evidence to suggest tissue specificity of effect which is very common in studies of splicing with 8/15 (53%) splicing factors showing associations with strain longevity in spleen, but only 3/15 (20%) in muscle. It would be interesting to determine whether the splicing events targeted by these sets of splicing factors also show associations between median strain lifespan and splice site usage, but such analysis would be very difficult due to degeneracy of splicing factor binding sites, potential for compensation by other splicing factors and the fact that splice site usage is dependent on the balance of enhancers and silencers rather than on the binding of a specific splicing factor *per se*. The pattern of longevity‐associated splicing regulator transcripts showed little overlap between the spleen and muscle data sets, with only changes to *Srsf3* expression being common to both tissues. This is in line with our previous findings, as we have previously shown that although fibroblasts and endothelial cells that have undergone *in vitro* senescence both show deregulation of splicing factors, the patterns of precisely which regulators are altered show quite marked differences between cell types (Holly *et al*., 2013). Spleen is a lymphoid organ, consisting of large numbers of white blood cells. Most of the transcripts extracted from spleen will arise from B cells, T cells and mononuclear phagocytes. Our findings may thus reflect the hypothesis that aging of the immune system is one of the drivers of development of aging phenotypes (Franceschi & Campisi, 2014). It should also be considered that the preponderance of splicing factor expression changes in spleen compared with muscle could also reflect an accelerated rate of aging and more extensive tissue modification in spleen compared to muscle. White blood cells are also highly heterogeneous, reactive and proliferative but relatively unspecialized compared to the highly differentiated nonproliferative muscle cells. It may be that muscle requires less adaptive response than spleen cells, since it has a defined and tightly regulated function with less need to respond to environmental challenge.

Previous work from our group has identified that offspring of long‐lived parents may have better health (Dutta *et al*., 2013a,b). In the current study, two of the associations between splicing factor expression and longevity were also seen in RNA samples derived from the peripheral blood of participants in the InCHIANTI study (Ferrucci *et al*., 2000), where we found relationships between expression of the *HNRNPA2B1* and *HNRNPA1* transcripts and parental longevity. *HNRNPA2B1* demonstrated increased expression in blood RNA from people with at least one long‐lived parent, whereas parental longevity (as a continuous trait reflecting the combined age at death of both parents) was associated with lower *HNRNPA1* expression (Table 1). *Hnrnpa1* is also downregulated with greater lifespan in mouse splenocytes, but the *HNRNPA2B1* effect in humans is reversed compared to what we observe in the mice. This may be because the association between *Hnrnpa2b1* expression and longevity is most marked in the young animals of the long‐lived strains, and our human subjects are mostly elderly, with a mean age of approximately 75 years (Harries *et al*., 2011). Interestingly, both *Hnrnpa2b1* and *Hnrnpa1* are known to be determinants of lifespan in *Drosophila* species, by virtue of their regulation of the TDB‐43 protein (Romano *et al*., 2014). TDB‐43 is crucial in fruit flies for correct splicing and regulation of mRNA stability and is associated with amyotrophic lobar sclerosis and frontotemporal lobar degeneration in humans (Neumann *et al*., 2007; Kim *et al*., 2013). Mutations have also been described in age‐related diseases such as Alzheimer's, Parkinson's and Huntington's diseases (Baloh, 2011). Recent studies have shown that the action of TDB‐43 relies on its ability to tether hnRNPA2B1 and hnRNPA1 proteins, and disruption or abolition of this association dramatically reduces lifespan in *Drosophila* (Romano *et al*., 2014).

The consequences of altered splicing in spleen give a broad picture of altered expression and processing of genes involved in reduced cell senescence, superior DNA repair and retained cellular proliferative capacity in the long‐lived strains. Old animals of long‐lived strains of mice expressed reduced amounts of *Cdkn2a* isoforms compared with old animals of average‐lived strains. *Cdkn2a* is an important marker of cellular senescence (Tominaga, 2015) and ablation of *Cdkn2a* expression reverses aging phenotypes in klotho mice (Sato *et al*., 2015), indicating that old animals of long‐lived strains may have lower levels of senescent cells. Young animals of longer‐lived strains also expressed profiles of *Trp53* isoforms consistent with enhanced transcription and cell growth properties compared to young animals of average‐lived strains, since they express higher levels of full‐length p53 and lower levels of truncated p53AS which is thought to have antagonistic function (Wu *et al*., 1997; Almog *et al*., 2000; Huang *et al*., 2002). Altered splicing of inflammatory genes involved in muscle remodelling produces a picture consistent with lower levels of pro‐inflammatory signalling by virtue of lower levels of *Il1b* and *Il6* expression in young animals and reduced *Tnf* signalling in the older animals of long‐lived strains.

We saw fewer associations of splicing factor expression with chronological age than we expected based on our previous human data. Our previous work suggests that splicing factor expression is strongly associated with age in humans and with cellular senescence in human cell models (Harries *et al*., 2011; Holly *et al*., 2013). In our human work, the per‐year age‐related changes in splicing factor expression were also relatively small (beta coefficients ranging from −0.01 to 0.005) (Holly *et al*., 2013), which may explain why strong effects were not seen in the current mouse study where our sample numbers were much smaller. There is also considerable interstrain heterogeneity for most splicing factors; the young animals of one strain may express lower basal levels of splicing factors than the older animals of another and effects of aging may thus be difficult to detect in small numbers of samples (Fig. S1). These data suggest that the associations of splicing factor expression with longevity across strains may actually be considerably larger than effects of age alone on splicing factor expression within strains. Again, most changes seen in this study were seen in spleen, which is consistent with a key role in senescence for immune‐mediated drivers of aging and aging phenotypes.

The limitations of our study include the relatively small sample sizes, restriction of our analyses to a small number of tissues, and the fact that we have assessed splicing patterns only at the mRNA level. Gene expression is a highly variable parameter in biological systems, so future experiments are likely to need larger sample sizes and assessment of potential effects in other tissue types, as well as assessment of effects on protein levels. Although the genes tested were selected *a priori* and therefore do not require adjustment for multiple testing, one must recognize that this does not entirely remove the possibility of false positives. It must also be considered that differences in splicing factor and isoform expression may arise not only from changes in the amount of transcription, but also from differences in the relative stabilities of different isoforms. These changes may form part of the mechanistic basis for our associations, as we would not expect stability changes unrelated to longevity to associate statistically with strain median lifespan. Finally, in the human follow up work described here, we were also restricted by the availability of expression data for all interesting splicing factors on the array and the likelihood that any effects were likely to be moderate on a per‐year basis as they were in our previous human age data. This is likely to have reduced our power in the human study, and thus further work in larger populations is now required to definitively explore this possibility in human subjects.

Another potential caveat is that the splicing factor expression differences we have discovered in this study reflect other strain differences that are unrelated to longevity. Whilst this is a possibility, the links between splicing factor expression and aging in humans and other animals are well documented from our previous work (Harries *et al*., 2011; Holly *et al*., 2013) and that of other groups (Meshorer & Soreq, 2002). Our observation that the lifespan‐associated expression changes relating to *Hnnrpa2b1* and *Hnrnpa1* we observe in mice are also translatable to humans is also supportive of our conclusions. More broadly, the importance of splicing factors in determination of lifespan is also suggested by studies of the effects of calorific restriction in mice (Swindell, 2009) and studies of the relationship between copy number variant (CNV) polymorphisms and longevity in humans (Glessner *et al*., 2013).

Some of the genetic differences between strains may actually contribute mechanistically to the differences in strain median lifespan. To that effect, we carried out a bioinformatic analysis on the potential for genetic variation discordant between strains to lead to gene regulation differences for *Hnrnpa2b1* and *Hnrnpa1*, where we also found effects in man. We discovered some minor changes to the bioinformatically predicted strength of SRSF2 and SRSF5 binding within *Hnrnpa2b1* and a potential amino acid change for an alternatively expressed isoform of *Hnrnpa1*. Although these predictions are interesting, the predicted effects of the changes on *Hnrnpa2b1* or *Hnrnpa1* expression or activity are likely to be slight. The splicing effects cause only a slight alteration to predicted binding efficiency of SRSF2 or SRSF5, and the coding change involves the substitution of a serine for a glycine in an alternatively spliced cassette exon of *Hnrnpa1,* which may not comprise the major isoform at this locus. These amino acids are in any case of similar size and charge and may not cause much change to protein functionality. It is likely the effects on median strain longevity arise from multiple changes in many genes.

To be able to assign definitive causality for a role for splicing factors as determinants of longevity, it would be necessary to carry out detailed functional experiments *in vivo* and *in vitro*, which could form the basis for future studies. Such studies could comprise constitutive or conditional knockout or overexpression studies in animal models followed by assessment of effects on lifespan, or *in vitro* manipulation of splicing factor levels followed by investigation of effects on cellular senescence. Such an approach has previously been employed for the *Hnrnpa1* and *Hnrnpa2b1* genes where upregulation of the *Hnrnpa1* gene or the *Hnrnpa2* isoform of the *Hnrnpa2b1* gene in mouse hepatocarcinoma cells was shown to cause activation of the RAS‐MAPK‐ERK pathway (Shilo *et al*., 2014). This is potentially important since a recent study has shown that activation of the RAS‐ERK‐ETS pathway is a key determinant of lifespan in Drosophila species (Slack *et al*., 2015).

Both *in vivo* and *in vitro* studies to moderate the levels or activity of splicing factors in relation to longevity would not be without caveat. Splice site choice is a complex phenomenon and relies upon the balance of splicing activators or silencers, rather than the activity of a single splicing factor *per se* (Cartegni *et al*., 2002). This finding, together with observations that exon and intron splicing enhancers and silencers often cluster near splice sites, raises the possibility of compensation between splicing regulatory factors. Selective modulation of a single splicing factor may not then show direct effects on longevity, effects would most probably only be noted after knockdown or overexpression of multiple splicing factors which would have technical challenges both *in vitro* and *in vivo*.

This study reports the first evidence of a link between expression of splicing regulator genes and strain lifespan in mice, together with data in support of potential roles for *HNRNPA1* and *HNRNPA2B1* in parental longevity in humans. We hypothesize that an influence of splicing factor expression on longevity may be mediated by slower immune aging and a protection from malignancy in the young mice of long‐lived strains by virtue of restricting expression of SR and hnRNP proteins which have oncogenic potential in young animals. Both of the splicing factor transcripts demonstrating reduced expression in the old long‐lived mice belonged to the hnRNP class of splicing regulators, indicating that these mice may have less inhibition of splice site usage and be better able to maintain splicing, and therefore cellular plasticity into older age. The changes in splicing factor expression in the spleen are accompanied by changes in alternatively spliced genes indicative of reduced cell senescence, superior DNA repair and retained cellular proliferative capacity, and changes in splicing factor expression in muscle are indicative of lower pro‐inflammatory signalling. Our data highlight the importance of regulation of mRNA processing in determination of lifespan and suggest that splicing factors may provide novel points of intervention for future therapies to reduce disease burden in old age. Moreover, since some of these strains (e.g. C57BL/6J, A/J) are commonly used for the creation and cross‐breeding of genetically modified mice, strain‐specific alterations in alternative splicing could account for unexpected contributions to the final phenotype arising from the genetic background.

## Experimental procedures

### Mouse strains used for analysis

Strains were chosen on the basis of differential lifespan (A/J, NOD.B10Sn‐H2^b^/J, PWD.Phj, 129S1/SvlmJ, C57BL/6J and WSB/EiJ; see Table 2 for lifespan details) that were measured in a longitudinal study (Yuan *et al*., 2009, 2011, 2012) at Jackson Laboratory Nathan Shock Center of Excellence in the Basic Biology of Aging. Strains with extremely short lifespans (median lifespan less than 600 days) were excluded on the basis that a short lifespan may be associated with significant comorbidities. Characteristics of the mice and the numbers of animals used in each category are given in Table 2. All mice used in this study were male. All tissues were obtained from the mice of a cross‐sectional study that was conducted at the same period of time and in the same mouse room with the longitudinal study. Animal housing conditions have been fully described previously (Yuan *et al*., 2009, 2012). Briefly, mice were fed *ad libitum* an autoclaved pellet diet with 6% fat and acidified water (pH 2.8–3.1). Animals were kept on a 12:12‐h light/dark cycle at 50% relative humidity at 21–23 °C, in a restricted access specific pathogen‐free barrier facility. Mice were housed four animals per pen in individually ventilated polycarbonate cages supplied with HEPA‐filtered air and were tested quarterly for (and were free of) common viral, bacterial and mycoplasmal species. Mice were inspected daily and excluded if ill. At 6 or 20/22 months of age, mice were euthanized by CO_2_ asphyxiation, followed by blood collection via cardiac puncture and cervical dislocation. A detailed description of the tissue collection procedure is given in Data S1. Immediately after death, spleen and quadriceps muscle tissues were excised and snap‐frozen in vapour‐phase liquid nitrogen for storage within 5 min of collection. Tissues were stored at −80 °C.

**Table 2:** Characteristics of mouse strains used in this study

Strain	Strain Median lifespan (days)^a^	Strain Max Age (days)	Longevity class	N Young	N Old
A/J	623	785	Average lifespan	Spleen – 7 Muscle – 8	Spleen – 7 Muscle – 7
NOD.B10Sn‐H2^b^/J	696	954	Average lifespan	Spleen – 4 Muscle – 4	Spleen – 6 Muscle – 6
PWD.PhJ	813	956	Average lifespan	Spleen – 5 Muscle – 4	Spleen – 6 Muscle – 6
129S1/SvlmJ	882	1044	Long‐lived	Spleen – 10 Muscle – 4	Spleen – 10 Muscle – 10
C57BL/6J	901	1061	Long‐lived	Spleen – 10 Muscle – 10	Spleen – 8 Muscle – 9
WSB/EiJ	1005	1213	Long‐lived	Spleen – 5 Muscle – 5	Spleen – 10 Muscle – 10

a Strain Max Age = the mean of the longest lived 20% within each strain. Data for median and maximum lifespans are given in Yuan *et al*. (2011) from a longitudinal study that was performed in conjunction with the cross‐sectional study described in the present paper.

The mean lifespan and the maximum lifespan (20% longest lived) are given for each strain used in this study. All mice used in this study were male. Young mice were 6 months old, and old mice were 20–22 months old. Muscle tissue was taken from the quadriceps.

### Splicing factor candidate genes for analysis

An *a priori* list of splicing factor candidate genes were chosen on the basis that they were associated with human aging in populations and in primary human cell lines that had undergone *in vitro* senescence in our previous work (Harries *et al*., 2011; Holly *et al*., 2013). The list of genes included the positive regulatory splicing factors *Srsf1, Srsf2, Srsf3, Srsf6, Srsf18* and *Tra2β*, the negative regulatory splicing inhibitors *Hnrnpa0, Hnrnpa1, Hnrnpa2b1, Hnrnpd, Hnrnph3, Hnrnpk, Hnrnpm, Hnrnpul2* and the *Sf3b1* subunit of the U2 spliceosome snRNP, which we have previously shown to be associated with age‐related altered DNA methylation (Holly *et al*., 2014). Assays were obtained in custom TaqMan low‐density array (TLDA) format from Life Technologies (Foster City, CA, USA). Assay Identifiers are given in Data S1.

### Alternatively spliced target genes in spleen

Genes were chosen for assessment of alternative splicing in spleen on the basis of potential roles in cellular senescence (*Cdkn2a*), cell cycle regulation (*Trp53*,* Myc*), extracellular matrix (*Fn1, Vcan*) or DNA damage response (*Atm, Chek2*), since these genes may also be important in determination of lifespan. We designed TaqMan quantitative real‐time PCR assays to identify specific isoforms or groups of isoforms (if large numbers of common regions rendered the design of specific probes impossible). Assays were obtained in custom TaqMan low‐density array (TLDA) format from Life Technologies. Assay Identifiers are given in Data S2. A list of transcripts captured by each assay are given in Data S3.

### Alternatively spliced target genes in muscle

Genes were selected for analysis of alternative splicing in muscle on the basis of potential roles in inflammatory processes relating to muscle remodelling since we have shown in our previous work that these processes are key determinants of muscle strength in older humans (Harries *et al*., 2012; Blackwell *et al*., 2015). Our gene list included isoforms of the *Il1b, Il6, Nfkb1, Stat1* and *Tnf* genes. As above, TaqMan quantitative real‐time PCR assays were designed to identify specific isoforms or groups of isoforms (if large numbers of common regions rendered the design of specific probes impossible). Assays were obtained in custom TaqMan low‐density array (TLDA) format from Life Technologies. Assay Identifiers are given in Data S2.

### RNA extraction and reverse transcription

Tissue samples were removed from storage and placed in 1 mL TRI Reagent^®^ solution supplemented with the addition of 10 mm MgCl_2_ to aid recovery of microRNAs for future analysis (Kim *et al*., 2012). Samples were then completely homogenized (15mins for spleen samples, 30mins for muscle samples) using a bead mill (Retsch Technology GmbH, Haan, Germany). Phase separation was carried out using chloroform. Total RNA was precipitated from the aqueous phase by means of an overnight incubation at −20 °C with isopropanol. RNA pellets were then ethanol‐washed twice and resuspended in RNase‐free dH2O. RNA quality and concentration was assessed by Nanodrop spectrophotometry (Wilmington, DE, USA). Complementary DNA (cDNA) was then reverse‐transcribed from 100 ng total RNA using the Invitrogen VILO cDNA synthesis kit (Life Technologies) in 20 μL reactions according to manufacturer's instructions.

### Quantitative real‐time PCR and data analysis

Quantitative RT–PCRs were performed on the ABI 7900HT platform (Life Technologies) on the TaqMan low‐density array (TLDA) platform. Cycling conditions were 50 °C for 2 min, 94.5 °C for 10 min and 50 cycles of 97 °C for 30 s and 57.9 °C for 1 min. The reaction mixes included 50 μL TaqMan^®^ Universal PCR Mastermix II (no AmpErase^®^ UNG) (Life Technologies), 30 μL dH2O and 20 μL cDNA template. 100 μL reaction solution was dispensed into the TLDA card chamber and centrifuged twice for 1 min at 216 × g to ensure distribution of solution to each well. The expression of transcripts in each sample was measured in duplicate replicates. The comparative Ct technique was used to calculate the expression of each test transcript (28). Expression was assessed relative to the global mean of expression and normalized to the median level of expression for each individual transcript. Data were log‐transformed to ensure normal distribution of data. Associations of transcript expression were assessed by linear regression against age, or lifespan as appropriate. Associations of transcript expression with mouse age were assessed in all animals and in animals of average‐lived or long‐lived strains individually, and associations of transcript expression with strain lifespan were assessed in all animals and in young and old groups individually. We also assessed the effect of potential nonlinearity of response for the splicing factor genes in spleen and muscle by a secondary analysis using binary logistic regression on data split by the median lifespan of all the strains. These statistical analyses were carried out using spss v.22 (IBM, North Castle, NY, USA). Interaction between strain longevity and mouse age was assessed using data categorized into average‐lived or long‐lived on the basis of interstrain median lifespan and was carried out in stata v.14 (StataCorp, College Station, TX, USA).

### Gene expression cluster analysis for heterogeneity of splicing factor expression with age

The expression of each gene was z‐transformed to be on the scale of standard deviations from the mean, and then the expression values for each gene were plotted against the corresponding sample to generate a heat map. Hierarchical clustering methods were used to group similar expression profiles together. This analysis was done using the ‘heatmap.2’ package in R statistical software package v3.1.1 (Vienna, Austria).

### Association between splicing factor expression and parental longevity in the InCHIANTI population

The participants in the InCHIANTI study aged 65+ years were categorized based on the age at death of their parents, the parental longevity score (PLS). Participants (total *n* = 405) were classified as either ‘two short‐lived parents’ (*n* = 17), ‘one short‐ and one intermediate‐lived parent’ (*n* = 140), ‘two intermediate‐lived parents’ (*n* = 190) or ‘any long‐lived parents’ (*n* = 58). Short‐, intermediate‐ and long‐lived cut‐offs were calculated separately for mothers and fathers based on the normal distribution of age at death in the cohort, as described in Dutta *et al*. (2013a). The cut‐offs for mothers were as follows: short‐lived (49–72 years), intermediate‐lived (72–95 years) and long‐lived (> 95 years); mothers aged < 49 years at death were classed as premature and excluded. The cut‐offs for fathers were: short‐lived (52–67 years), intermediate‐lived (68–89 years) and long‐lived (> 89 years); fathers aged < 52 years at death were classed as premature and excluded.

To assess the association between the gene expression levels of the 15 splicing factor genes in whole blood as defined in our previous work (Holly *et al*., 2013) and parental longevity score, linear regression models were carried out using R statistical software package v3.1.1 (Vienna, Austria), with gene expression as the dependent variable. Models were adjusted for age, sex, waist circumference, highest education level attained, smoking (pack‐years), study site, batches and cell counts (neutrophils, monocytes, basophils, eosinophils and whole white blood cell count). Gene expression data were rank‐normalized prior to analysis to remove any skew.

### Bioinformatic assessment of potential regulatory effects of genetic variation within Hnrnpa2b1 and Hnrnpa1 genes

To assess the potential for genetic variation to contribute to splicing factor expression differences that we observe between strains, we have carried out a detailed examination of the strain‐discordant genetic differences in the splicing factor genes *Hnrnpa2b1* and *Hnrnpa1,* which demonstrate links with longevity in both mouse and man. Complete genome sequence data were available for 4 of the strains we have used in our analysis (C57BL/6J, 129S1/SvImJ, A/J and WSB/EiJ). SNPs discordant between strains were examined for evidence of effects on gene regulation by a variety of bioinformatic approaches. Firstly, SNPs located in the 5′ UTR of the *Hnrnpa2b1* or *Hnrnpa1* genes were assessed for position relative to known transcription factor binding sites using regrna2.0 (http://regrna2.mbc.nctu.edu.tw/), an integrated web server tool that allows screening for potential regulatory elements. Secondly, intronic SNPs were assessed for their ability to interrupt exon and intron splicing enhancer and silencer loci using regrna2.0 and esefinder (http://rulai.cshl.edu/cgi-bin/tools/ESE3/esefinder.cgi?process=home), a specific tool for the identification of splicing regulatory elements. Finally, sequences in the 3′ untranslated region were screened for ability to disrupt elements with potential to disrupt elements important for mRNA stability (A‐rich elements, C to U RNA editing sites and miRNA binding sites) using regrna2.0.

## Author contributions

BL carried out laboratory work, analysis and contributed to the manuscript, LCP contributed the statistical assessment of parental longevity in the human samples. FE contributed technical assistance and analysis. KF advised on selection and mouse strains and analytical approaches. RY and LP designed and managed the initial mouse lifespan study, identified genetic differences in *Hnrnpa2b1* and *Hnrnpa1* discordant between strains and contributed to the manuscript. DEH codirected the Jackson laboratory Nathan Shock Centre of Excellence in the Basic Biology of Aging at the time this work was performed, and was instrumental in facilitating the mouse collection and animal husbandry facilities used in this study. GAK contributed to and reviewed the manuscript. LF provided access to the InCHIANTI study data. DM comanaged the study and reviewed the manuscript. LWH managed the study, interpreted the data and wrote the manuscript.

## Supporting information


**Fig. S1** Interstrain heterogeneity of splicing factor expression according to mouse age in mouse strains of different lifespan.

**Table S1** Splicing factor expression in mouse spleen tissue by lifespan, across 6 strains of different longevities.

**Table S2** Splicing factor expression in mouse muscle tissue by lifespan, across 6 strains of different longevities.

**Table S3** Alternative isoform expression in mouse spleen tissue by lifespan, across 6 strains of different longevities.

**Table S4** Alternative isoform expression in mouse muscle tissue by lifespan, across 6 strains of different longevities.

**Table S5** Splicing factor expression in mouse spleen tissue by age in young (6 months) and old (20–22 months) mice.

**Table S6** Splicing factor expression in mouse muscle tissue by age in young (6 months) and old (20–22 months) mice.

**Table S7** Alternative isoform expression in mouse spleen tissue by age in young (6 months) and old (20–22 months) mice.

**Table S8** Alternative isoform expression in mouse muscle tissue by age in young (6 months) and old (20–22 months) mice.

**Table S9** Analyses of potential interactions between mouse strain longevity and mouse age.

**Table S10** Splicing factor expression in mouse spleen tissue by lifespan, across 6 strains of different longevities by binary logistic regression.

**Table S11** Splicing factor expression in mouse muscle tissue by lifespan, across 6 strains of different longevities by binary logistic regression.

**Table S12** Genetic variation within the mouse Hnrnpa2b1 and Hnrnpa1 genes and its predicted effect on gene regulation.

**Data S1** Detailed tissue collection protocol.

**Data S2** Assay identifiers and sequence details for qRT–PCR assays used in this study.

**Data S3** Alternatively expressed isoforms captured by quantitative real‐time PCR assays. Microsoft word file. Additional information describing precisely which alternatively expressed isoforms of analysed genes are captured by each probe set.
