# Supplementary material for: Changes in the expression of splicing factor transcripts and variations in alternative splicing are associated with lifespan in mice and humans
Source: Aging Cell. 2016 Jun 30;15(5):903–13. doi: 10.1111/acel.12499 (PMC5013025; doi:10.1111/acel.12499)
Supplement: Supplementary file 1 — Fig. S1 Interstrain heterogeneity of splicing factor expression according to mouse age in mouse strains of different lifespan. [file ACEL-15-903-s001.docx]

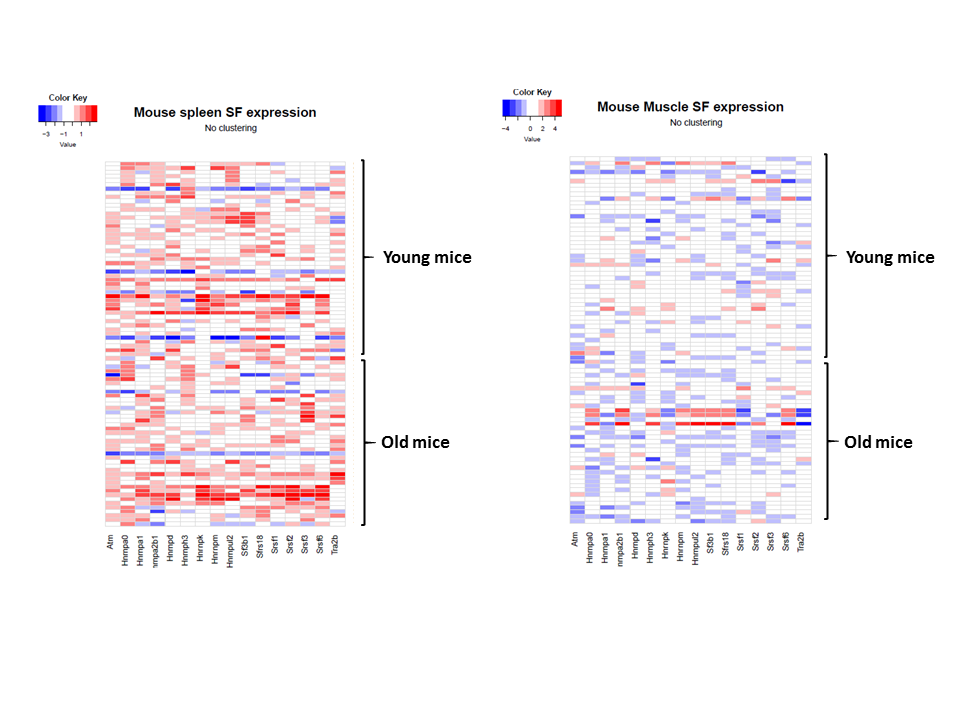


**Additional figure 1: Heat maps demonstrating inter- and intra-strain heterogeneity in splicing factor expression by age**. Splicing factor expression is given along the bottom of each heat map. Mouse age is given on the Y-axis. No clustering of expression signatures in young or old mice is noted. Data are Z-scored, and each transcript is expressed as standard deviation from the mean.
