# Supplementary material for: Changes in the expression of splicing factor transcripts and variations in alternative splicing are associated with lifespan in mice and humans
Source: Aging Cell. 2016 Jun 30;15(5):903–13. doi: 10.1111/acel.12499 (PMC5013025; doi:10.1111/acel.12499)
Supplement: Supplementary file 2 — Table S1 Splicing factor expression in mouse spleen tissue by lifespan, across 6 strains of different longevities. [file ACEL-15-903-s002.docx]

**Additional table 1: Splicing factor expression in mouse spleen tissue by lifespan, across 6 strains of different longevities. Data from mice of all ages, young mice only (6 months) and old mice only (20-22 months) are given separately**. Data with statistically-significant effects at <0.05 are given in bold, underlined italic text. *Tra2β* was not expressed in PWD/Phj mice so this strain was excluded from the analysis for this marker. P values were determined from linear regression of logged data.

|  | **All Ages** | | | **Young mice only** | | | **Old mice only** | | |
| --- | --- | --- | --- | --- | --- | --- | --- | --- | --- |
| **Gene** | **Beta coefficient** | **Std Error** | **P value** | **Beta coefficient** | **Std Error** | **P value** | **Beta coefficient** | **Std Error** | **P value** |
| ***Hnrnpa0*** | 0.175 | 0.01 | 0.10 | 0.297 | 0.02 | 0.06 | 0.075 | 0.02 | 0.62 |
| ***Hnrnpa1*** | -0.151 | 0.01 | 0.16 | -0.396 | 0.01 | ***0.01*** | 0.061 | 0.01 | 0.69 |
| ***Hnrnpa2b1*** | -0.256 | 0.01 | ***0.02*** | -0.578 | 0.01 | ***<0.0001*** | 0.036 | 0.01 | 0.81 |
| ***Hnrnpd*** | -0.021 | 0.01 | 0.85 | 0.020 | 0.12 | 0.90 | -0.045 | 0.02 | 0.76 |
| ***Hnrnph3*** | -0.133 | 0.01 | 0.22 | -0.193 | 0.01 | 0.23 | -0.098 | 0.02 | 0.513 |
| ***Hnrnpk*** | -0.316 | 0.01 | ***0.003*** | -0.288 | 0.02 | 0.07 | -0.337 | 0.02 | ***0.02*** |
| ***Hnrnpm*** | -0.310 | 0.01 | ***0.003*** | -0.307 | 0.01 | 0.051 | -0.312 | 0.01 | ***0.03*** |
| ***Hnrnpul2*** | -0.223 | 0.01 | ***0.04*** | -0.214 | 0.01 | 0.18 | -0.231 | 0.01 | 0.12 |
| ***Sf3b1*** | -0.212 | 0.01 | ***0.05*** | -0.267 | 0.01 | 0.09 | -0.174 | 0.01 | 0.24 |
| ***Srsf18*** | 0.062 | 0.01 | 0.57 | 0.041 | 0.02 | 0.80 | 0.073 | 0.02 | 0.63 |
| ***Srsf1*** | -0.092 | 0.01 | 0.39 | -0.253 | 0.02 | 0.11 | 0.047 | 0.02 | 0.76 |
| ***Srsf2*** | -0.208 | 0.01 | 0.052 | -0.224 | 0.02 | 0.16 | -0.189 | 0.02 | 0.20 |
| ***Srsf3*** | -0.130 | 0.01 | 0.23 | -0.362 | 0.02 | ***0.02*** | 0.125 | 0.01 | 0.40 |
| ***Srsf6*** | -0.157 | 0.01 | 0.14 | -0.132 | 0.02 | 0.41 | -0.176 | 0.01 | 0.24 |
| ***Tra2β*** | -0.122 | 0.01 | 0.26 | -0.361 | 0.01 | ***0.02*** | 0.081 | 0.01 | 0.59 |
