# Supplementary material for: Changes in the expression of splicing factor transcripts and variations in alternative splicing are associated with lifespan in mice and humans
Source: Aging Cell. 2016 Jun 30;15(5):903–13. doi: 10.1111/acel.12499 (PMC5013025; doi:10.1111/acel.12499)
Supplement: Supplementary file 3 — Table S2 Splicing factor expression in mouse muscle tissue by lifespan, across 6 strains of different longevities. [file ACEL-15-903-s003.docx]

**Additional table 2: Splicing factor expression in mouse muscle tissue by lifespan across 6 strains of different longevities. Data from mice of all ages, young mice only (6 months) and old mice only (20-22 months) are given separately**. Data with statistically-significant effects at <0.05 are given in underlined, bold, italic text. *Tra2β *was not expressed in PWD/Phj mice so this strain was excluded from the analysis for this marker. P values were determined from linear regression of logged data.

|  | **All Ages** | | | **Young mice only** | | | **Old mice only** | | |
| --- | --- | --- | --- | --- | --- | --- | --- | --- | --- |
| **Gene** | **Beta coefficient** | **Std Error** | **P value** | **Beta coefficient** | **Std Error** | **P value** | **Beta coefficient** | **Std Error** | **P value** |
| ***Hnrnpa0*** | 0.124 | 0.02 | 0.27 | -0.086 | 0.03 | 0.62 | 0.357 | 0.02 | ***0.01*** |
| ***Hnrnpa1*** | -0.093 | 0.01 | 0.40 | -0.200 | 0.01 | 0.25 | -0.037 | 0.01 | 0.80 |
| ***Hnrnpa2b1*** | 0.005 | 0.02 | 0.96 | -0.166 | 0.03 | 0.34 | 0.231 | 0.02 | 0.11 |
| ***Hnrnpd*** | -0.238 | 0.01 | ***0.03*** | -0.177 | 0.01 | 0.31 | -0.321 | 0.01 | ***0.03*** |
| ***Hnrnph3*** | -0.048 | 0.02 | 0.67 | -0.227 | 0.03 | 0.19 | 0.115 | 0.02 | 0.44 |
| ***Hnrnpk*** | -0.146 | 0.01 | 0.19 | -0.030 | 0.02 | 0.86 | -0.239 | 0.02 | 0.10 |
| ***Hnrnpm*** | -0.019 | 0.01 | 0.86 | -0.211 | 0.02 | 0.23 | 0.168 | 0.01 | 0.25 |
| ***Hnrnpul2*** | -0.050 | 0.02 | 0.66 | -0.223 | 0.03 | 0.20 | 0.152 | 0.02 | 0.30 |
| ***Sf3b1*** | -0.165 | 0.02 | 0.14 | -0.298 | 0.03 | 0.08 | -0.008 | 0.02 | 0.96 |
| ***Srsf18*** | -0.180 | 0.01 | 0.10 | -0.315 | 0.03 | 0.07 | -0.047 | 0.02 | 0.75 |
| ***Srsf1*** | 0.147 | 0.02 | 0.19 | 0.209 | 0.03 | 0.23 | 0.092 | 0.02 | 0.54 |
| ***Srsf2*** | -0.177 | 0.01 | 0.11 | -0.264 | 0.01 | 0.13 | -0.134 | 0.01 | 0.36 |
| ***Srsf3*** | -0.093 | 0.01 | 0.40 | -0.396 | 0.01 | ***0.02*** | 0.105 | 0.01 | 0.48 |
| ***Srsf6*** | -0.066 | 0.02 | 0.55 | -0.136 | 0.03 | 0.44 | 0.023 | 0.02 | 0.88 |
| ***Tra2β*** | 0.030 | 0.03 | 0.80 | 0.054 | 0.05 | 0.77 | 0.013 | 0.03 | 0.94 |
