# Supplementary material for: Changes in the expression of splicing factor transcripts and variations in alternative splicing are associated with lifespan in mice and humans
Source: Aging Cell. 2016 Jun 30;15(5):903–13. doi: 10.1111/acel.12499 (PMC5013025; doi:10.1111/acel.12499)
Supplement: Supplementary file 4 — Table S3 Alternative isoform expression in mouse spleen tissue by lifespan, across 6 strains of different longevities. [file ACEL-15-903-s004.docx]

**Additional table 3: Alternative isoform expression in mouse spleen tissue by lifespan across 6 strains of different longevities. Data from mice of all ages, young (6 months) and old (24 months) are given separately**. UCSC transcript Identities identified by each probe set are given under the gene names. Data with statistically-significant effects at <0.05 are given in bold, underlined italic text. P values were determined from linear regression on logged data.

|  | **All mice** | | | **Young mice only** | | | **Old mice only** | | |
| --- | --- | --- | --- | --- | --- | --- | --- | --- | --- |
| **Isoform** | **Beta coefficient** | **Std Error** | **P value** | **Beta coefficient** | **Std Error** | **P value** | **Beta coefficient** | **Std Error** | **P value** |
| **Atm-1,3**  uc009pme.2  uc009pmd.2 | 0.038 | 0.01 | 0.73 | 0.151 | 0.05 | 0.35 | -0.054 | 0.01 | 0.71 |
| **Atm-2**  uc012gtj.1 | -0.001 | 0.01 | 0.99 | 0.148 | 0.02 | 0.36 | -0.162 | 0.01 | 0.27 |
| **Cdkn2a-1**  Uc008toi.1 | -0.164 | 0.02 | 0.13 | 0.186 | 0.02 | 0.26 | -0.433 | 0.03 | ***0.002*** |
| **Cdkn2a-2**  uc008toh.1 | -0.247 | 0.02 | ***0.02*** | 0.108 | 0.02 | 0.51 | -0.590 | 0.02 | ***<0.0001*** |
| **Chek2-1**  uc008yrw.1 | -0.102 | 0.02 | 0.34 | -0.281 | 0.02 | 0.08 | 0.070 | 0.02 | 0.63 |
| **Chek2-2**  uc008yrx.1 | -0.066 | 0.01 | 0.54 | -0.292 | 0.02 | 0.07 | 0.154 | 0.02 | 0.29 |
| **Fn1-1**  uc007bju.2 | 0.253 | 0.01 | ***0.02*** | 0.349 | 0.02 | ***0.03*** | 0.166 | 0.02 | 0.25 |
| **Fn1-2,5**  uc007bjv.2  uc007bjy.2 | -0.044 | 0.02 | 0.68 | -0.030 | 0.02 | 0.86 | -0.064 | 0.03 | 0.66 |
| **Lmna-1**  uc008pvj.3 | -0.022 | 0.02 | 0.84 | -0.070 | 0.03 | 0.67 | 0.033 | 0.02 | 0.82 |
| **Lmna-1,3**  uc008pvj.3  uc008pvl.3 | -0.114 | 0.02 | 0.29 | -0.237 | 0.02 | 0.14 | 0.017 | 0.02 | 0.91 |
| **Myc-1**  uc007vyh.2 | -0.098 | 0.01 | 0.36 | -0.242 | 0.01 | 0.13 | 0.000 | 0.01 | 0.99 |
| **Myc-1,2,3**  uc007vyh.2  uc007vyg.2  uc007vyi.1 | -0.124 | 0.01 | 0.25 | -0.243 | 0.02 | 0.13 | -0.037 | 0.01 | 0.80 |
| **Trp53-1,3,4**  uc007jql.2  uc007jqm.2  uc007jqn.2 | 0.144 | 0.02 | 0.18 | 0.406 | 0.01 | ***0.009*** | -0.025 | 0.01 | 0.86 |
| **Trp53-2**  uc011xww.1 | -0.236 | 0.01 | ***0.03*** | -0.250 | 0.01 | 0.12 | -0.214 | 0.07 | 0.14 |
| **Trp53-3**  uc007jqm.2 | -0.230 | 0.01 | ***0.03*** | -0.352 | 0.01 | ***0.03*** | -0.121 | 0.01 | 0.41 |
| **Vcan-1**  uc007rjg.1 | 0.365 | 0.02 | ***0.001*** | 0.570 | 0.03 | ***<0.0001*** | 0.243 | 0.04 | 0.09 |
| **Vcan-2**  uc011zck.1 | 0.109 | 0.04 | 0.422 | -0.058 | 0.05 | 0.77 | 0.220 | 0.06 | 0.24 |
