# Supplementary material for: Changes in the expression of splicing factor transcripts and variations in alternative splicing are associated with lifespan in mice and humans
Source: Aging Cell. 2016 Jun 30;15(5):903–13. doi: 10.1111/acel.12499 (PMC5013025; doi:10.1111/acel.12499)
Supplement: Supplementary file 5 — Table S4 Alternative isoform expression in mouse muscle tissue by lifespan, across 6 strains of different longevities. [file ACEL-15-903-s005.docx]

**Additional table 4: Alternative isoform expression in mouse muscle tissue by lifespan across 6 strains of different longevities. Data from mice of all ages, young (6 months) and old (20-22 months) are given separately**. Data with statistically-significant effects at <0.05 are given in bold, underlined italic text. P values were determined from linear regression of logged data.

|  | **All mice** | | | **Young mice only** | | | **Old mice only** | | |
| --- | --- | --- | --- | --- | --- | --- | --- | --- | --- |
| **Isoform** | **Beta coefficient** | **Std Error** | **P value** | **Beta coefficient** | **Std Error** | **P value** | **Beta coefficient** | **Std Error** | **P value** |
| **Il1b-2**  uc008mht.1 | -0.193 | 0.03 | 0.09 | -0.458 | 0.04 | ***0.007*** | -0.053 | 0.04 | 0.73 |
| **Il1b-2,3**  uc008mht.1  uc008mhu.1 | -0.138 | 0.03 | 0.23 | -0.306 | 0.04 | 0.08 | -0.074 | 0.03 | 0.63 |
| **IL6-1,2**  uc008wuu.1  uc008wuv.1 | -0.225 | 0.03 | 0.052 | -0.482 | 0.04 | ***0.006*** | -0.100 | 0.04 | 0.52 |
| **Il6-1,3**  uc008wuu.1  uc008wuw.1 | -0.279 | 0.03 | ***0.01*** | -0.268 | 0.05 | 0.13 | -0.281 | 0.04 | 0.06 |
| **Nfkb1-1,4,5**  uc008rly.1  uc012cyg.1  uc008rlx.1 | 0.264 | 0.01 | ***0.02*** | 0.370 | 0.01 | ***0.03*** | 0.186 | 0.01 | 0.22 |
| **Nfkb1-1,5**  uc008rly.1  uc008rlx.1 | 0.076 | 0.02 | 0.51 | -0.071 | 0.03 | 0.69 | 0.294 | 0.01 | ***0.05*** |
| **Nfkb1-2**  uc012cyf.1 | 0.160 | 0.02 | 0.16 | 0.082 | 0.02 | 0.65 | 0.213 | 0.02 | 0.16 |
| **Nfkb1-3,4,5**  uc008rlw.1  uc012cyg.1  uc008rlx.1 | 0.268 | 0.01 | ***0.02*** | 0.283 | 0.01 | 0.11 | 0.275 | 0.01 | 0.07 |
| **Nfkb1-4,5**  uc012cyg.1  uc008rlx.1 | 0.313 | 0.01 | ***0.005*** | 0.257 | 0.02 | 0.15 | 0.371 | 0.01 | ***0.01*** |
| **Stat1-1**  uc007axy.1 | -0.066 | 0.03 | 0.57 | 0.069 | 0.05 | 0.70 | -0.171 | 0.04 | 0.26 |
| **Stat1-3,4**  uc007axz.1  uc007aya.2 | -0.472 | 0.01 | ***<0.0001*** | -0.234 | 0.02 | 0.19 | -0.625 | 0.01 | ***<0.0001*** |
| **Stat1-2,3,4,5**  uc007ayd.2  uc007axz.1  uc007aya.2  uc007ayb.2 | 0.088 | 0.01 | 0.44 | 0.241 | 0.01 | 0.18 | -0.014 | 0.01 | 0.92 |
| **Stat1-2,4,5,6**  uc007ayd.2  uc007aya.2  uc007ayb.2  uc007ayc.2 | 0.219 | 0.01 | 0.052 | 0.429 | 0.01 | ***0.01*** | 0.096 | 0.01 | 0.53 |
| **Stat1-5**  uc007ayb.2 | 0.332 | 0.01 | ***0.005*** | 0.378 | 0.02 | ***0.03*** | 0.299 | 0.02 | ***0.04*** |
| **Stat1-6**  uc007ayc.2 | 0.338 | 0.03 | ***0.003*** | 0.460 | 0.03 | ***0.007*** | 0.271 | 0.04 | 0.08 |
| **Tnf-1,2**  uc008cgr.2  uc012arb.2 | -0.269 | 0.02 | ***0.02*** | -0.282 | 0.03 | 0.11 | -0.256 | 0.02 | 0.09 |
| **Tnf-1,3**  uc008cgr.2  uc008cgs.2 | -0.233 | 0.02 | ***0.04*** | -0.183 | 0.03 | 0.31 | -0.280 | 0.02 | 0.06 |
| **Tnf-3**  uc008cgs.2 | -0.177 | 0.02 | 0.121 | -0.139 | 0.03 | 0.44 | -0.199 | 0.03 | 0.19 |
