# Supplementary material for: Changes in the expression of splicing factor transcripts and variations in alternative splicing are associated with lifespan in mice and humans
Source: Aging Cell. 2016 Jun 30;15(5):903–13. doi: 10.1111/acel.12499 (PMC5013025; doi:10.1111/acel.12499)
Supplement: Supplementary file 6 — Table S5 Splicing factor expression in mouse spleen tissue by age in young (6 months) and old (20–22 months) mice. [file ACEL-15-903-s006.docx]

**Additional table 5: Splicing factor expression in mouse spleen tissue by age in young (6 months) and old (20-22 months) mice. Data from mice of all strains, average-lived strains (median age <847.5 days) and long-lived (median lifespan >847.5 days) strains are given separately**. Data with statistically-significant effects at <0.05 are given in bold, underlined italic text. *Tra2β* was not expressed in PWD/Phj mice so this strain was excluded from the analysis for this marker. P values were determined from linear regression analysis of logged data.

|  | **All strains** | | | **Average-lived strains only** | | | **Long-lived strains only** | | |
| --- | --- | --- | --- | --- | --- | --- | --- | --- | --- |
| **Gene** | **Beta coefficient** | **Std Error** | **P value** | **Beta coefficient** | **Std Error** | **P value** | **Beta coefficient** | **Std Error** | **P value** |
| ***Hnrnpa0*** | -0.031 | 0.04 | 0.77 | 0.018 | 0.05 | 0.92 | -0.053 | 0.05 | 0.70 |
| ***Hnrnpa1*** | -0.015 | 0.03 | 0.89 | -0.278 | 0.04 | 0.11 | 0.134 | 0.03 | 0.34 |
| ***Hnrnpa2b1*** | -0.035 | 0.04 | 0.74 | -0.460 | 0.05 | ***0.005*** | 0.248 | 0.04 | 0.07 |
| ***Hnrnpd*** | 0.038 | 0.03 | 0.73 | -0.043 | 0.06 | 0.81 | 0.094 | 0.04 | 0.50 |
| ***Hnrnph3*** | -0.170 | 0.03 | 0.11 | -0.278 | 0.06 | 0.11 | -0.09 | 0.04 | 0.54 |
| ***Hnrnpk*** | 0.012 | 0.04 | 0.91 | 0.081 | 0.07 | 0.64 | -0.104 | 0.03 | 0.46 |
| ***Hnrnpm*** | -0.034 | 0.03 | 0.75 | -0.108 | 0.05 | 0.54 | -0.001 | 0.04 | 0.99 |
| ***Hnrnpul2*** | 0.018 | 0.03 | 0.87 | -0.042 | 0.06 | 0.81 | 0.059 | 0.04 | 0.68 |
| ***Sf3B1*** | 0.000 | 0.03 | 0.99 | -0.073 | 0.05 | 0.68 | 0.05 | 0.04 | 0.72 |
| ***Srsf18*** | 0.158 | 0.04 | 0.14 | 0.098 | 0.06 | 0.58 | 0.193 | 0.06 | 0.17 |
| ***Srsf1*** | -0.157 | 0.04 | 0.14 | -0.341 | 0.06 | ***0.05*** | -0.042 | 0.05 | 0.76 |
| ***Srsf2*** | -0.178 | 0.04 | 0.10 | -0.266 | 0.07 | 0.12 | -0.150 | 0.04 | 0.284 |
| ***Srsf3*** | -0.215 | 0.04 | ***0.04*** | -0.449 | 0.07 | ***0.007*** | -0.007 | 0.04 | 0.96 |
| ***Srsf6*** | -0.082 | 0.04 | 0.45 | -0.124 | 0.06 | 0.48 | -0.068 | 0.03 | 0.63 |
| ***Tra2β*** | -0.221 | 0.03 | 0.053 | -0.526 | 0.06 | ***0.008*** | -0.050 | 0.04 | 0.73 |
