# Supplementary material for: Changes in the expression of splicing factor transcripts and variations in alternative splicing are associated with lifespan in mice and humans
Source: Aging Cell. 2016 Jun 30;15(5):903–13. doi: 10.1111/acel.12499 (PMC5013025; doi:10.1111/acel.12499)
Supplement: Supplementary file 7 — Table S6 Splicing factor expression in mouse muscle tissue by age in young (6 months) and old (20–22 months) mice. [file ACEL-15-903-s007.docx]

**Additional table 6: Splicing factor expression in mouse muscle tissue by age in young (6 months) and old (20-22 months) mice. Data from mice of all strains, average-lived strains (mean lifespan <847.5 days) and long-lived strains (mean lifespan >847.5 days) are given separately**. Data with statistically-significant effects at <0.05 are given in bold, underlined italic text. *Tra2β* was not expressed in PWD/Phj mice so this strain was excluded from the analysis for this marker. P values were determined from linear regression of logged data.

|  | **All Ages** | | | **Average-lived strains only** | | | **Long-lived strains only** | | |
| --- | --- | --- | --- | --- | --- | --- | --- | --- | --- |
| **Gene** | **Beta coefficient** | **Std Error** | **P value** | **Beta coefficient** | **Std Error** | **P value** | **Beta coefficient** | **Std Error** | **P value** |
| ***Hnrnpa0*** | 0.123 | 0.05 | 0.27 | -0.057 | 0.08 | 0.75 | 0.236 | 0.07 | 0.11 |
| ***Hnrnpa1*** | 0.217 | 0.03 | ***0.05*** | 0.174 | 0.04 | 0.32 | 0.267 | 0.05 | 0.07 |
| ***Hnrnpa2b1*** | -0.048 | 0.06 | 0.67 | -0.228 | 0.08 | 0.19 | 0.075 | 0.07 | 0.61 |
| ***Hnrnpd*** | 0.123 | 0.03 | 0.27 | 0.221 | 0.04 | 0.20 | 0.113 | 0.04 | 0.44 |
| ***Hnrnph3*** | -0.001 | 0.06 | 0.99 | -0.184 | 0.08 | 0.29 | 0.122 | 0.08 | 0.41 |
| ***Hnrnpk*** | -0.046 | 0.04 | 0.68 | 0.086 | 0.07 | 0.62 | -0.128 | 0.05 | 0.39 |
| ***Hnrnpm*** | 0.187 | 0.04 | 0.09 | 0.040 | 0.05 | 0.82 | 0.281 | 0.05 | 0.053 |
| ***Hnrnpul2*** | 0.029 | 0.05 | 0.80 | -0.134 | 0.07 | 0.44 | 0.128 | 0.08 | 0.39 |
| ***Sf3B1*** | 0.017 | 0.06 | 0.88 | -0.146 | 0.08 | 0.40 | 0.127 | 0.08 | 0.39 |
| ***Srsf18*** | 0.095 | 0.05 | 0.39 | -0.055 | 0.07 | 0.76 | 0.222 | 0.07 | 0.13 |
| ***Srsf1*** | -0.046 | 0.06 | 0.68 | 0.016 | 0.10 | 0.93 | -0.114 | 0.07 | 0.44 |
| ***Srsf2*** | 0.084 | 0.03 | 0.45 | 0.134 | 0.04 | 0.44 | 0.083 | 0.05 | 0.58 |
| ***Srsf3*** | 0.100 | 0.04 | 0.37 | -0.086 | 0.05 | 0.62 | 0.272 | 0.04 | 0.06 |
| ***Srsf6*** | -0.100 | 0.06 | 0.37 | -0.159 | 0.08 | 0.36 | -0.063 | 0.09 | 0.67 |
| ***Tra2β*** | -0.06 | 0.10 | 0.61 | -0.018 | 0.18 | 0.93 | -0.082 | 0.13 | 0.58 |
