# Supplementary material for: Changes in the expression of splicing factor transcripts and variations in alternative splicing are associated with lifespan in mice and humans
Source: Aging Cell. 2016 Jun 30;15(5):903–13. doi: 10.1111/acel.12499 (PMC5013025; doi:10.1111/acel.12499)
Supplement: Supplementary file 8 — Table S7 Alternative isoform expression in mouse spleen tissue by age in young (6 months) and old (20–22 months) mice. [file ACEL-15-903-s008.docx]

**Additional table 7: Alternative isoform expression in mouse spleen tissue by age in young (6 months) and old (20 -22 months) mice. Data from mice of all strains, average-lived strains (mean lifespan <847.5 days) and long-lived strains (mean lifespan (>847 days) are given separately**. UCSC transcript Identities identified by each probe set are given under the gene names. Data with statistically-significant effects at <0.05 are given in bold, underlined italic text. P values were determined from linear regression of logged data.

|  | **All strains** | | | **Average-lived strains only** | | | **Long-lived strains only** | | |
| --- | --- | --- | --- | --- | --- | --- | --- | --- | --- |
| **Isoform** | **Beta coefficient** | **Std Error** | **P value** | **Beta coefficient** | **Std Error** | **P value** | **Beta coefficient** | **Std Error** | **P value** |
| **Atm-1,3**  uc009pme.2  uc009pmd.2 | -0.044 | 0.04 | 0.69 | 0.127 | 0.06 | 0.47 | -0.156 | 0.05 | 0.36 |
| **Atm-2**  uc012gtj.1 | -0.072 | 0.04 | 0.50 | 0.163 | 0.06 | 0.35 | -0.277 | 0.05 | 0.10 |
| **Cdkn2a-1**  Uc008toi.1 | 0.399 | 0.06 | ***<0.0001*** | 0.659 | 0.09 | ***<0.0001*** | 0.224 | 0.08 | 0.10 |
| **Cdkn2a-2**  uc008toh.1 | 0.529 | 0.06 | ***<0.0001*** | 0.815 | 0.06 | ***<0.0001*** | 0.346 | 0.08 | ***0.007*** |
| **Chek2-1**  uc008yrw.1 | -0.331 | 0.05 | ***0.002*** | -0.499 | 0.07 | ***0.002*** | -0.227 | 0.07 | 0.10 |
| **Chek2-2**  uc008yrx.1 | -0.349 | 0.05 | ***0.001*** | -0.542 | 0.06 | ***0.001*** | -0.232 | 0.06 | 0.10 |
| **Fn1-1**  uc007bju.2 | 0.065 | 0.07 | 0.55 | 0.164 | 0.08 | 0.35 | -0.004 | 0.06 | 0.98 |
| **Fn1-2,5**  uc007bjv.2  uc007bjy.2 | 0.105 | 0.05 | 0.33 | 0.167 | 0.06 | 0.34 | 0.086 | 0.08 | 0.54 |
| **Lmna-1**  uc008pvj.3 | -0.047 | 0.06 | 0.66 | -0.147 | 0.11 | 0.40 | 0.047 | 0.06 | 0.73 |
| **Lmna-1,3**  uc008pvj.3  uc008pvl.3 | -0.198 | 0.05 | 0.06 | -0.335 | 0.09 | ***0.05*** | -0.101 | 0.06 | 0.47 |
| **Myc-1**  uc007vyh.2 | 0.03 | 0.03 | 0.76 | -0.027 | 0.07 | 0.88 | 0.109 | 0.03 | 0.43 |
| **Myc-1,2,3**  uc007vyh.2  uc007vyg.2  uc007vyi.1 | 0.062 | 0.04 | 0.56 | 0.000 | 0.08 | 0.99 | 0.155 | 0.03 | 0.26 |
| **Trp53-1,3,4**  uc007jql.2  uc007jqm.2  uc007jqn.2 | -0.243 | 0.02 | ***0.02*** | 0.007 | 0.03 | 0.97 | -0.407 | 0.02 | ***0.002*** |
| **Trp53-2**  uc011xww.1 | -0.114 | 0.02 | 0.29 | -0.06 | 0.03 | 0.73 | -0.147 | 0.03 | 0.29 |
| **Trp53-3**  uc007jqm.2 | -0.240 | 0.03 | ***0.03*** | -0.313 | 0.05 | 0.07 | -0.194 | 0.04 | 0.16 |
| **Vcan-1**  uc007rjg.1 | 0.063 | 0.09 | 0.56 | 0.198 | 0.11 | 0.26 | 0.004 | 0.13 | 0.98 |
| **Vcan-2**  uc011zck.1 | -0.034 | 0.13 | 0.80 | -0.217 | 0.21 | 0.31 | 0.129 | 0.15 | 0.47 |
