# Supplementary material for: Changes in the expression of splicing factor transcripts and variations in alternative splicing are associated with lifespan in mice and humans
Source: Aging Cell. 2016 Jun 30;15(5):903–13. doi: 10.1111/acel.12499 (PMC5013025; doi:10.1111/acel.12499)
Supplement: Supplementary file 9 — Table S8 Alternative isoform expression in mouse muscle tissue by age in young (6 months) and old (20–22 months) mice. [file ACEL-15-903-s009.docx]

**Additional table 8: Alternative isoform expression in mouse muscle tissue by age in young (6 months) and old (20 -22 months) mice. Data from mice of all strains, average-lived strains (mean lifespan <847.5 days) and long-lived strains (mean lifespan (>847 days) are given separately**. UCSC transcript Identities identified by each probe set are given under the gene names. Data with statistically-significant effects at <0.05 are given in bold, underlined italic text. P values were determined from linear regression of logged data.

|  | **All strains** | | | **Average-lived mice only** | | | **Long-lived mice only** | | |
| --- | --- | --- | --- | --- | --- | --- | --- | --- | --- |
| **Isoform** | **Beta coefficient** | **Std Error** | **P value** | **Beta coefficient** | **Std Error** | **P value** | **Beta coefficient** | **Std Error** | **P value** |
| **Il1b-2**  uc008mht.1 | 0.373 | 0.10 | ***0.001*** | 0.139 | 0.17 | 0.44 | 0.601 | 0.10 | ***<0.0001*** |
| **Il1b-2,3**  uc008mht.1  uc008mhu.1 | 0.393 | 0.10 | ***<0.0001*** | 0.231 | 0.15 | 0.20 | 0.544 | 0.11 | ***<0.0001*** |
| **IL6-1,2**  uc008wuu.1  uc008wuv.1 | 0.230 | 0.11 | ***0.05*** | -0.008 | 0.20 | 0.97 | 0.435 | 0.13 | ***0.003*** |
| **Il6-1,3**  uc008wuu.1  uc008wuw.1 | -0.196 | 0.12 | 0.09 | -0.247 | 0.18 | 0.17 | -0.161 | 0.16 | 0.30 |
| **Nfkb1-1,4,5**  uc008rly.1  uc012cyg.1  uc008rlx.1 | 0.015 | 0.03 | 0.90 | 0.066 | 0.05 | 0.71 | -0.050 | 0.04 | 0.74 |
| **Nfkb1-1,5**  uc008rly.1  uc008rlx.1 | 0.083 | 0.06 | 0.45 | -0.110 | 0.06 | 0.54 | 0.161 | 0.09 | 0.29 |
| **Nfkb1-2**  uc012cyf.1 | -0.070 | 0.06 | 0.54 | -0.149 | 0.08 | 0.41 | -0.029 | 0.08 | 0.85 |
| **Nfkb1-3,4,5**  uc008rlw.1  uc012cyg.1  uc008rlx.1 | -0.088 | 0.03 | 0.44 | -0.165 | 0.04 | 0.36 | -0.070 | 0.05 | 0.64 |
| **Nfkb1-4,5**  uc012cyg.1  uc008rlx.1 | -0.107 | 0.04 | 0.35 | -0.170 | 0.07 | 0.34 | -0.091 | 0.05 | 0.55 |
| **Stat1-1**  uc007axy.1 | 0.040 | 0.11 | 0.73 | 0.171 | 0.18 | 0.34 | -0.071 | 0.14 | 0.64 |
| **Stat1-3,4**  uc007axz.1  uc007aya.2 | -0.105 | 0.05 | 0.36 | 0.221 | 0.04 | 0.22 | -0.207 | 0.07 | 0.17 |
| **Stat1-2,3,4,5**  uc007ayd.2  uc007axz.1  uc007aya.2  uc007ayb.2 | -0.053 | 0.04 | 0.64 | 0.064 | 0.05 | 0.73 | -0.130 | 0.05 | 0.39 |
| **Stat1-2,4,5,6**  uc007ayd.2  uc007aya.2  uc007ayb.2  uc007ayc.2 | -0.203 | 0.03 | 0.07 | -0.014 | 0.04 | 0.94 | -0.321 | 0.05 | ***0.03*** |
| **Stat1-5**  uc007ayb.2 | -0.059 | 0.05 | 0.61 | 0.242 | 0.04 | 0.17 | -0.020 | 0.07 | 0.89 |
| **Stat1-6**  uc007ayc.2 | -0.121 | 0.09 | 0.30 | -0.139 | 0.15 | 0.44 | -0.155 | 0.11 | 0.32 |
| **Tnf-1,2**  uc008cgr.2  uc012arb.2 | -0.058 | 0.06 | 0.61 | 0.040 | 0.08 | 0.83 | -0.082 | 0.09 | 0.59 |
| **Tnf-1,3**  uc008cgr.2  uc008cgs.2 | 0.053 | 0.06 | 0.64 | 0.178 | 0.09 | 0.32 | 0.002 | 0.08 | 0.99 |
| **Tnf-3**  uc008cgs.2 | -0.076 | 0.07 | 0.51 | 0.145 | 0.10 | 0.42 | -0.219 | 0.09 | 0.15 |
