# Supplementary material for: Changes in the expression of splicing factor transcripts and variations in alternative splicing are associated with lifespan in mice and humans
Source: Aging Cell. 2016 Jun 30;15(5):903–13. doi: 10.1111/acel.12499 (PMC5013025; doi:10.1111/acel.12499)
Supplement: Supplementary file 10 — Table S9 Analyses of potential interactions between mouse strain longevity and mouse age. [file ACEL-15-903-s010.docx]

**Additional table 9: Analyses of potential interactions between mouse strain longevity and mouse age**. Std. Err = standard error, 95% Ci = 95% Confidence intervals. Average lived strains have a lifespan of <847.5 days, long-lived strains have a mean lifespan of >847.5 days. Young mice are 6 months, old mice are 20-22 months old. Statistically signifciant results are indicated in bold underlined text.

| **Longevity:age interactions - splicing factors**  **(Spleen)** | | | | |
| --- | --- | --- | --- | --- |
| **Gene** | **Beta coefficient** | **Std. Err** | **95% CI** | **P value** |
| *Hnrnpa1*  Average-lived/old  Long-lived/young  Long-lived/old | -0.06  -0.14  -0.09 | 0.04  0.04  0.04 | -0.14 to 0.18  -0.21 to -0.56  -0.02 to 0.02 | 0.13  **0.001**  **0.01** |
| *Hnrnpa2b1*  Average-lived/old  Long-lived/young  Long-lived/old | -0.15  -0.19  -0.12 | 0.05  0.05  0.05 | -0.26 to -0.05  -0.29 to -0.10  -0.21 to -0.02 | **0.005**  **<0.0001**  **0.02** |
| *Hnrnpk*  Average-lived/old  Long-lived/young  Long-lived/old | 0.03  -0.18  -0.20 | 0.05  0.05  0.05 | -0.07 to 0.13  -0.27 to -0.08  -0.29 to -0.10 | 0.52  **<0.0001**  **<0.0001** |
| *Hnrnpm*  Average-lived/old  Long-lived/young  Long-lived/old | -0.03  -0.15  -0.14 | 0.05  0.05  0.05 | -0.12 to 0.06  -0.23 to -0.05  -0.23 to -0.08 | 0.52  **0.002**  **0.001** |
| *Hnrnpul2*  Average-lived/old  Long-lived/young  Long-lived/old | -0.01  -0.08  -0.06 | 0.05  0.05  0.05 | 0.09 to -0.12  0.02 to -0.18  0.03 to -0.16 | 0.80  0.11  0.20 |
| *Sf3b1*  Average-lived/old  Long-lived/young  Long-lived/old | -0.02  -0.11  -0.09 | 0.05  0.05  0.05 | -0.12 to 0.07  -0.20 to -0.02  -0.19 to -0.01 | 0.64  **0.02**  **0.04** |
| *Srsf3*  Average-lived/old  Long-lived/young  Long-lived/old | -0.19  -0.17  -0.17 | 0.05  0.05  0.05 | -0.30 to -0.08  -0.28 to -0.07  -0.28 to -0.08 | **0.001**  **0.001**  **0.001** |
| *Tra2β*  Average-lived/old  Long-lived/young  Long-lived/old | -0.18  -0.13  -0.14 | 0.06  0.05  0.05 | -0.30 to -0.07  -0.23 to -0.26  -0.24 to -0.04 | **0.002**  **0.02**  **0.01** |
| **Longevity:age interactions - splicing factors**  **(Muscle)** | | | | |
| **Gene** | **Beta coefficient** | **Std. Err** | **95% CI** | **P value** |
| *Srsf3*  Average-lived/old  Long-lived/young  Long-lived/old | -0.03  -0.14  -0.05 | 0.05  0.05  0.05 | -0.13 to 0.07  -0.24 to -0.03  -0.14 to 0.04 | 0.61  **0.01**  0.28 |
| **Longevity:age interactions - Isoforms**  **(Spleen)** | | | | |
| **Gene** | **Beta coefficient** | **Std. Err** | **95% CI** | **P value** |
| *Cdkn2a-1*  Average-lived/old  Long-lived/young  Long-lived/old | 0.44  0.09  0.22 | 0.10  0.09  0.09 | 0.25 to 0.63  -0.09 to 0.27  0.06 to 0.40 | **<0.0001**  0.34  **0.01** |
| *Cdkn2a-2*  Average-lived/old  Long-lived/young  Long-lived/old | 0.50  0.04  0.27 | 0.09  0.08  0.08 | 0.32 to 0.67  -0.12 to 0.20  0.11 to 0.43 | **<0.0001**  0.66  **0.001** |
| *Chek2-1*  Average-lived/old  Long-lived/young  Long-lived/old | -0.24  -0.15  -0.26 | 0.08  0.07  0.07 | -0.39 to -0.08  -0.30 to 0.001  -0.40 to -0.12 | **0.003**  **0.05**  **<0.0001** |
| *Trp53-134*  Average-lived/old  Long-lived/young  Long-lived/old | 0.001  0.08  0.007 | 0.03  0.03  0.03 | -0.06 to 0.06  0.03 to 0.14  -0.05 to 0.06 | 0.97  **0.004**  0.79 |
| *Trp53-3*  Average-lived/old  Long-lived/young  Long-lived/old | -0.09  -0.06  -0.11 | 0.04  0.04  0.04 | -0.18 to 0.01  -0.15 to 0.03  -0.19 to -0.03 | 0.06  0.18  **0.01** |
| **Longevity:age interactions - Isoforms**  **(Muscle)** | | | | |
| **Gene** | **Beta coefficient** | **Std. Err** | **95% CI** | **P value** |
| *Il1b-2*  Average-lived/old  Long-lived/young  Long-lived/old | 0.13  -0.42  0.10 | 0.14  0.14  0.13 | -0.15 to 0.41  -0.70 to -0.14  -0.17 to 0.36 | 0.36  **0.004**  0.452 |
| *Il6-2*  Average-lived/old  Long-lived/young  Long-lived/old | -0.01  -0.36  0.05 | 0.17  0.17  0.15 | -0.35 to 0.33  -0.70 to -0.02  -0.25 to 0.36 | 0.96  **0.04**  0.72 |
| *Stat1-34*  Average-lived/old  Long-lived/young  Long-lived/old | 0.05  -0.07  -0.17 | 0.07  0.07  0.06 | -0.08 to 0.18  -0.20 to 0.06  -0.29 to -0.05 | 0.44  0.28  **0.01** |
| *Stat1-2456*  Average-lived/old  Long-lived/young  Long-lived/old | -0.003  0.11  0.003 | 0.05  0.05  0.05 | -0.10 to 0.10  0.12 to 0.21  -0.09 to 0.09 | 0.95  **0.03**  0.951 |
| *Tnf-2*  Average-lived/old  Long-lived/young  Long-lived/old | 0.02  -0.18  -0.23 | 0.09  0.09  0.08 | -0.16 to 0.20  -0.16 to 0.001  -0.39 to -0.06 | 0.85  0.06  **0.008** |
