# Supplementary material for: Changes in the expression of splicing factor transcripts and variations in alternative splicing are associated with lifespan in mice and humans
Source: Aging Cell. 2016 Jun 30;15(5):903–13. doi: 10.1111/acel.12499 (PMC5013025; doi:10.1111/acel.12499)
Supplement: Supplementary file 11 — Table S10 Splicing factor expression in mouse spleen tissue by lifespan, across 6 strains of different longevities by binary logistic regression. [file ACEL-15-903-s011.docx]

**Additional table 10: Splicing factor expression in mouse spleen tissue by lifespan, across 6 strains of different longevities by binary logistic regression. Data from mice of all ages, young mice only (6 months) and old mice only (20-22 months) are given separately**. Data with statistically-significant effects at <0.05 are given in bold, underlined italic text. *Tra2β* was not expressed in PWD/Phj mice so this strain was excluded from the analysis for this marker. P values were determined from binary logistic regression of logged data. P values marked by stars are also significant in linear regression analysis.

|  | **All Ages** | | | **Young mice only** | | | **Old mice only** | | |
| --- | --- | --- | --- | --- | --- | --- | --- | --- | --- |
| **Gene** | **Beta coefficient** | **Std Error** | **P value** | **Beta coefficient** | **Std Error** | **P value** | **Beta coefficient** | **Std Error** | **P value** |
| ***Hnrnpa0*** | 1.80 | 1.31 | 0.17 | 2.27 | 1.95 | 0.24 | 1.40 | 1.78 | 0.43 |
| ***Hnrnpa1*** | -5.78 | 2.05 | ***0.005*** | -10.40 | 3.63 | ***0.004**** | -2.50 | 2.53 | 0.32 |
| ***Hnrnpa2b1*** | -2.89 | 1.47 | ***0.05**** | -10.23 | 3.36 | ***0.002**** | 1.38 | 1.91 | 0.47 |
| ***Hnrnpd*** | -0.90 | 1.42 | 0.54 | -3.11 | 1.28 | 0.26 | -0.06 | 1.62 | 0.98 |
| ***Hnrnph3*** | 1.00 | 1.43 | 0.49 | -1.19 | 2.85 | 0.67 | 1.83 | 1.76 | 0.30 |
| ***Hnrnpk*** | -9.50 | 2.28 | ***<0.0001**** | -9.42 | 7.05 | ***0.008*** | -9.55 | 2.99 | ***0.001**** |
| ***Hnrnpm*** | -8.23 | 2.34 | ***<0.0001**** | -10.67 | 6.72 | ***0.01*** | -6.70 | 5.49 | ***0.02**** |
| ***Hnrnpul2*** | -2.88 | 3.37 | 0.07* | -4.70 | 2.95 | 0.09 | -1.84 | 1.90 | 0.33 |
| ***Sf3b1*** | -4.79 | 1.81 | ***0.008**** | -8.00 | 3.44 | ***0.02*** | -3.17 | 2.11 | 0.13 |
| ***Srsf18*** | -1.96 | 1.18 | 0.10 | -2.57 | 1.79 | 0.15 | -1.47 | 1.62 | 0.36 |
| ***Srsf1*** | -2.45 | 1.36 | 0.07 | -5.28 | 2.46 | ***0.03*** | -0.766 | 1.76 | 0.66 |
| ***Srsf2*** | -5.96 | 1.63 | ***<0.0001*** | -8.01 | 3.03 | ***0.008**** | -5.18 | 2.09 | ***0.01*** |
| ***Srsf3*** | -2.61 | 1.35 | 0.054 | -6.16 | 2.35 | ***0.009*** | 0.49 | 2.03 | 0.81 |
| ***Srsf6*** | -6.07 | 1.98 | ***0.002*** | -6.90 | 3.11 | ***0.03*** | -5.55 | 2.63 | ***0.04*** |
| ***Tra2β*** | -1.67 | 1.72 | 0.33 | -7.48 | 3.32 | ***0.02**** | 1.95 | 2.29 | 0.39 |
