# Supplementary material for: Changes in the expression of splicing factor transcripts and variations in alternative splicing are associated with lifespan in mice and humans
Source: Aging Cell. 2016 Jun 30;15(5):903–13. doi: 10.1111/acel.12499 (PMC5013025; doi:10.1111/acel.12499)
Supplement: Supplementary file 12 — Table S11 Splicing factor expression in mouse muscle tissue by lifespan, across 6 strains of different longevities by binary logistic regression. [file ACEL-15-903-s012.docx]

**Additional table 11: Splicing factor expression in mouse muscle tissue by lifespan across 6 strains of different longevities by binary logistic regression analysis. Data from mice of all ages, young mice only (6 months) and old mice only (20-22 months) are given separately**. Data with statistically-significant effects at <0.05 are given in underlined, bold, italic text. *Tra2β *was not expressed in PWD/Phj mice so this strain was excluded from the analysis for this marker. P values were determined from binary logistic regression of logged data. P values marked by stars are also significant in linear regression analysis.

|  | **All Ages** | | | **Young mice only** | | | **Old mice only** | | |
| --- | --- | --- | --- | --- | --- | --- | --- | --- | --- |
| **Gene** | **Beta coefficient** | **Std Error** | **P value** | **Beta coefficient** | **Std Error** | **P value** | **Beta coefficient** | **Std Error** | **P value** |
| ***Hnrnpa0*** | 2.20 | 1.11 | ***0.05*** | 0.33 | 1.21 | 0.79 | 6.08 | 2.30 | ***0.008**** |
| ***Hnrnpa1*** | -2.39 | 1.60 | 0.13 | -3.70 | 2.48 | 0.14 | -1.91 | 2.22 | 0.39 |
| ***Hnrnpa2b1*** | 0.66 | 0.95 | 0.49 | -0.48 | 1.11 | 0.62 | 3.60 | 2.01 | 0.07 |
| ***Hnrnpd*** | -6.27 | 2.09 | ***0.003**** | -4.29 | 2.64 | 0.10 | -9.40 | 3.45 | ***0.006**** |
| ***Hnrnph3*** | 0.16 | 0.89 | 0.86 | -1.06 | 1.31 | 0.42 | 1.44 | 1.33 | 0.23 |
| ***Hnrnpk*** | -4.52 | 1.47 | ***0.002*** | -2.55 | 1.90 | 0.18 | -6.99 | 2.49 | ***0.005*** |
| ***Hnrnpm*** | -0.02 | 1.42 | 0.99 | -1.55 | 1.90 | 0.41 | 2.15 | 2.50 | 0.39 |
| ***Hnrnpul2*** | 0.08 | 0.94 | 0.93 | -0.79 | 1.19 | 0.51 | 1.67 | 1.64 | 0.31 |
| ***Sf3b1*** | -0.71 | 0.89 | 0.43 | -1.34 | 1.25 | 0.28 | 0.34 | 1.55 | 0.83 |
| ***Srsf18*** | -1.76 | 1.06 | 0.10 | -2.63 | 1.68 | 0.12 | -1.05 | 1.62 | 0.52 |
| ***Srsf1*** | 0.94 | 0.88 | 0.29 | 1.16 | 1.19 | 0.33 | 0.72 | 1.34 | 0.59 |
| ***Srsf2*** | -3.32 | 1.62 | ***0.04*** | -4.02 | 2.74 | 0.14 | -3.14 | 2.03 | 0.12 |
| ***Srsf3*** | -2.99 | 1.52 | ***0.05*** | -7.90 | 3.24 | ***0.02**** | -0.95 | 1.82 | 0.61 |
| ***Srsf6*** | -0.39 | 0.79 | 0.63 | -0.43 | 1.03 | 0.68 | -0.22 | 1.27 | 0.86 |
| ***Tra2β*** | -0.04 | 0.58 | 0.95 | 0.10 | 0.69 | 0.88 | -0.28 | 1.05 | 0.79 |
