# Supplementary material for: Changes in the expression of splicing factor transcripts and variations in alternative splicing are associated with lifespan in mice and humans
Source: Aging Cell. 2016 Jun 30;15(5):903–13. doi: 10.1111/acel.12499 (PMC5013025; doi:10.1111/acel.12499)
Supplement: Supplementary file 13 — Table S12 Genetic variation within the mouse Hnrnpa2b1 and Hnrnpa1 genes and its predicted effect on gene regulation. [file ACEL-15-903-s013.docx]

**Additional table 12: Genetic variation within the mouse *Hnrnpa2b1* and *Hnrnpa1* genes and its predicted effect on gene regulation**. This table gives the SNP identifiers, chromosomal location, gene position and predicted effect on amino acid sequence or parameters of gene regulation for the mouse *Hnrnpa2b1* and *Hnrnpa1* genes. 5’ UTR = 5’ untranslated region, I = intron, C = coding, SRE = splicing regulatory element, 3’ UTR = 3’ untranslated region. ARE = A-rich element, C>U = C to U RNA editing site, miR = microRNA binding, TF = transcription factor binding. Where 2 alternative gene position is given, this is due to the possibility that the SNP is located in different genetic regions for different isoforms. ‘/ ‘= not applicable

| **SNP name** | **Chromosomal location** | **Position in gene** | **TF** | **Coding change** | **SRE** | **ARE** | **C>U** | **miR** |
| --- | --- | --- | --- | --- | --- | --- | --- | --- |
| ***Hnrnpa2b1*** | | | | | | | | |
| rs46028062 | 6:51461052 | 3’UTR | / | / | / | NO | NO | NO |
| rs48993060 | 6:51465149 | I  3’UTR | /  / | /  / | NO  / | /  NO | /  NO | /  NO |
| rs224637794 | 6:51461566 | I  3’UTR | /  / | /  / | NO  / | /  NO | /  NO | /  NO |
| rs234970481 | 6:51465354 | I | / | / | NO | / | / | / |
| rs229198021 | 6:51465605 | I | / | / | NO | / | / | / |
| rs250280372 | 6:51465935 | I | / | / | NO | / | / | / |
| rs226722250 | 6:51466015 | I | / | / | NO | / | / | / |
| rs47522479 | 6:51466888 | I | / | / | NO | / | / | / |
| rs51031918 | 6:51467084 | I | / | / | **POSSIBLE** | / | / | / |
| rs252413833 | 6:51467157 | I | / | / | **POSSIBLE** | / | / | / |
| rs235452001 | 6:51469308 | I | / | / | NO | / | / | / |
| rs239268432 | 6:51469567 | I | / | / | NO | / | / | / |
| rs228820180 | 6:51469765 | 5’UTR | NO | / | / | / | / | / |
| rs257262812 | 6:51470336 | Intergenic | NO | / | / | / | / | / |
| ***Hnrnpa1*** | | | | | | | | |
| rs32398879 | 15:103242334 | I | / | / | NO | / | / | / |
| rs50030666 | 15:103242939 | I  C | /  / | /  **Gly257Ser** | NO  / | /  / | /  / | /  / |
