# Supplementary material for: Changes in the expression of splicing factor transcripts and variations in alternative splicing are associated with lifespan in mice and humans
Source: Aging Cell. 2016 Jun 30;15(5):903–13. doi: 10.1111/acel.12499 (PMC5013025; doi:10.1111/acel.12499)
Supplement: Supplementary file 14 — Data S1 Detailed tissue collection protocol. [file ACEL-15-903-s014.docx]

**Additional information file 1**

**Tissue collection procedure for the mouse strain comparison study**

Mice were housed in duplex "shoebox" cages. Two to three days prior to sacrifice and tissue collection, mouse cages were moved from the mouse room to the procedure room. All the mice from one pen (n = 4–5 mice) were sacrificed within 1–6 minutes of initial disturbance of the home cage. The mice in the remaining pen of the home cage were returned to the mouse room and sacrificed 2–4 weeks later. Each mouse to be sacrificed was removed from its cage and immediately euthanized by CO_2_ asphyxiation. As soon as the mouse stopped breathing and did not exhibit a reflex to a foot pinch, it was bled by cardiac puncture. The liver, spleen, kidneys and heart were removed in that order. Next, the skin sample, thigh muscle and thymus were removed. Portions of liver, spleen, kidney, skin, heart, and skeletal muscle were placed into RNAlater (SigmaAldrich) and immediately frozen in liquid nitrogen; the remainder of each tissue was placed in Cryo-tubes and frozen directly in liquid nitrogen. Four technicians participated in the dissection of each mouse to minimize the time from death to freezing the tissue (less than 2 minutes for liver, and less than 3 minutes for each of the remaining tissues). All tissues were stored at –80° C.
