# Supplementary material for: Changes in the expression of splicing factor transcripts and variations in alternative splicing are associated with lifespan in mice and humans
Source: Aging Cell. 2016 Jun 30;15(5):903–13. doi: 10.1111/acel.12499 (PMC5013025; doi:10.1111/acel.12499)
Supplement: Supplementary file 15 — Data S2 Assay identifiers and sequence details for qRT–PCR assays used in this study. [file ACEL-15-903-s015.docx]

| **Additional Information File 2**  **Splicing factors** |  |  |  |  |
| --- | --- | --- | --- | --- |
| Transcript | TaqMan® Assay | |  |  |
| Hnrnpa0 | Mm03809085_s1 | |  |  |
| Hnrnpa1 | Mm01303205_g1 | |  |  |
| Hnrnpa2b1 | Mm01325931_g1 | |  |  |
| Hnrnpd | Mm01201314_m1 | |  |  |
| Hnrnph3 | Mm01032120_g1 | |  |  |
| Hnrnpk | Mm01349462_m1 | |  |  |
| Hnrnpm | Mm00513070_m1 | |  |  |
| Hnrnpul2 | Mm01230949_m1 | |  |  |
| Sf3b1 | Mm00473100_m1 | |  |  |
| Sfrs18 | Mm01219239_m1 | |  |  |
| Srsf1 | Mm00557620_m1 | |  |  |
| Srsf2 | Mm00448705_m1 | |  |  |
| Srsf3 | Mm00786953_s1 | |  |  |
| Srsf6 | Mm00471475_m1 | |  |  |
| Tra2b | Mm00833637_mH | |  |  |
|  |  |  |  |  |
| **Spleen** |  |  |  |  |
| Isoform Target(s) - UCSC ID | **Assay Name** | Forward Primer | Reverse Primer | Probe |
| uc009pme.2 ; uc009pmd.2 | **ATM_13** | CGACCTGGGTTTGCATTGG | GTGCTAGACTCATGGTTTAAGATTTCAGA | CCTCACCGCTGCATTC |
| uc012gtj.1 | **ATM_2** | TGCTCTGCAGTGTCTAAGAAACAG | TGCCCTTACTCAACTCTTCAACTTC | TTTCACCCTGGCATATCG |
| uc008toi.1 | **CDKN2A_1** | GCCGCACCGGAATCCT | AAGAGCTGCTACGTGAACGT | CCCATCATCATCACCTGGTC |
| uc008toh.1 | **CDKN2A_2** | CAACGCCCCGAACTCTTTC | AAGAGCTGCTACGTGAACGT | CCCGATTCAGGTGATGAT |
| uc008yrw.1 | **CHEK2_1** | AAGAGACGAATACATCATGTCAAAAACTCT | CACTTTCTGACATGTCTTCCTCTCAA | ACACGCACCACTTCCA |
| uc008yrx.1 | **CHEK2_2** | TGAGTAACAACTCTGAAATCGCACTT | CACTTTCTGACATGTCTTCCTCTCAA | ACACGCACCACTTTAT |
| uc007bju.2 | **FN1_1** | CCCTACTACACTGACACAGCAA | GTGTCTGGACCGATATTGGTGAAT | ACGGCTGTCCCTCCTC |
| uc007bjv.2 ; uc007bjy.2 | **FN1_25** | AGCCCCTGATTGGGAGGAA | GTCATACCCAGGGTTGGTGAT | AAGACAGTTCAAAAGACCC |
| uc008pvj.3 | **LMNA_1** | GACGACGAGGATGGAGAAGAG | CGTGAGCGCAGGTTGTACT | CCGTGGTTCCCACTGCA |
| uc008pvj.3 ; uc008pvl.3 | **LMNA_13** | GTGCGTGAGGAGTTCAAGGA | CTGCGCAGCCAACAAGTC | AAGGCTCGCAACACCA |
| uc008pvk.3 | **LMNA_2** | AAGGCCTTGCTCTCTCTGG | CTGCGCAGCCAACAAGTC | CTTGGTGTTGCGGCCCT |
| uc007vyh.2 | **MYC_1** | GGATTTCCTTTGGGCGTTGGA | GGTCATAGTTCCTGTTGGTGAAGTT | AACCCCGACAGCCACG |
| uc007vyh.2 ; uc007vyg.2 ; uc007vyi.1 | **MYC_123** | CTAGTGCTGCATGAGGAGACA | ACAGACACCACATCAATTTCTTCCT | CAGCGACTCTGAAGAAG |
| uc007jql.2 ; uc007jqm.2 ; uc007jqn.2 | **TRP53_134** | GCAGGGTGTCACGCTTCT | TCCGACTGTGACTCCTCCAT | CAGTCATCCAGTCTTCG |
| uc011xww.1 | **TRP53_2** | GCAGGGTGTCACGCTTCT | GCTTCAGGCTTTTCTTGGATTTTCT | ACTGGCCGCTTCTC |
| uc007jqm.2 | **TRP53_3** | GTTAAAGGATGCCCATGCTACAGA | AGTTTGGGCTTTCCTCCTTGATC | TCCAGCCTCCAGCCTAG |
| uc007rjg.1 | **VCAN_1** | CCAAGTTCCACCCTGACATAAATGT | GGATGACCACTTACAATCATATCACTCA | ATCGACCTGTCTTGTTTTC |
| uc011zck.1 ; uc007rji.2 | **VCAN_23** | CCAAGTTCCACCCTGACATAAATGTTTATATTAT | CGTTGAGGCATGGGTTTGTTTTG | ACAGGACCTGATCTCTG |
|  |  |  |  |  |
| **Muscle** |  |  |  |  |
| Isoform Target(s) - UCSC ID | **Assay Name** | Forward Primer | Reverse Primer | Probe |
| uc012cdt.1 | **IL1B_1** | TGAAAGCTCTCCACCTCAATGG | GCTCATGGAGAATATCACTGGAGAAA | TCAACCAACAAGACTCCTC |
| uc008mht.1 | **IL1B_2** | GTTCCTGAACTCAACTGTGAAATGC | CGTCAACTTCAAAGAACAGGTCATT | TCATCACTGTCAAAAGGTG |
| uc008mht.1 ; uc008mhu.1 | **IL1B_23** | GACAGTGATGAGAATGACCTGTTCT | AGCCCAGGTCAAAGGTTTGG | AAGCAGCCCTTCATCTTT |
| uc008wuu.1 ; uc008wuv.1 | **IL6_12** | TCAATTCCAGAAACCGCTATGAAGT | GTCCCAAGAAGGCAACTGGAT | TCTGCAAGAGACTTCC |
| uc008wuu.1 ; uc008wuw.1 | **IL6_13** | GCCAGAGTCCTTCAGAGAGATACA | GCTTATCTGTTAGGAGAGCATTGGA | TCAACCAAGAGGTAAAAGA |
| uc008rly.1 ; uc012cyg.1 ; uc008rlx.1 | **NFKB1_145** | GCATTCTGACCTTGCCTATCTACAA | CCTGGCGGATGATCTCCTT | CTCTGTCTGTGAGTTGCC |
| uc008rly.1 ; uc008rlx.1 | **NFKB1_15** | GAGCCTCTAGTGAGAAGAACAAGAA | TTTGCAGGCCCCACATAGTT | ACAGGTCAAAATTTGC |
| uc012cyf.1 | **NFKB1_2** | CTGCTCCTTCTAAAACTCTCATGGA | TCTCCACACCACTGTCACAGA | CCCGGAGTTCATCTCAT |
| uc008rlw.1 ; uc012cyg.1 ; uc008rlx.1 | **NFKB1_345** | TCTGCCTCTCTCGTCTTCCT | TCTCCACACCACTGTCACAGA | CCCGGAGTTCATCTATG |
| uc012cyg.1 | **NFKB1_4** | GAGCCTCTAGTGAGAAGAACAAGAA | GCCCCACATAGTTGCAAATCTG | CAGGTCAAAAGGCCCC |
| uc012cyg.1 ; uc008rlx.1 | **NFKB1_45** | GGAAACTAGTGAACCGAAACCCTTT | GCGTTTCCTTTGCACTTCCT | CCCTGAAATCAAAGACAAAG |
| uc007axy.1 | **STAT1_1** | GGAGCTGGACAGTAAAGTCAGAAAT | CTCTTCGCCACACCATTGG | TCACCTTCATGACTTGATCC |
| uc007axy.1 ; uc007axz.1 ; uc007aya.2 | **STAT1_134** | CCTGCGTGCAGTGAGTGA | GCCGGCTCAGGGTATGG | CTGAAACGACTGGCTCTCA |
| uc007ayd.2 ; uc007axz.1 ; uc007aya.2 ; uc007ayb.2 | **STAT1_2345** | CTCTTAGCTTTGAAACCCAGTTGTG | AGATCACCACGACAGGAAGAGA | TTGACCTGGAGACCACC |
| uc007ayd.2 ; uc007aya.2 ; uc007ayb.2 ; uc007ayc.2 | **STAT1_2456** | CGAACTGGATACATCAAGACTGAGT | GTTGTCTGTGGTCTGAAGTCTAGAA | CTGTGTCTGAAGTCCACCC |
| uc007ayb.2 | **STAT1_5** | GCAGAGAGATTTGCCCAGACT | GCCGGCTCAGGGTATGG | CAGAGCTGAAACGATCACT |
| uc007ayc.2 | **STAT1_6** | CTCTTATCCTGCCGTTCTCACTTC | GCCGGCTCAGGGTATGG | TAGGTCGTTTCAGCTCTGC |
| uc008cgr.2 ; uc012arb.2 | **TNF_12** | CACGCTCTTCTGTCTACTGAACTT | CTGATGAGAGGGAGGCCATTTG | AAGGGATGAGAAGTTCC |
| uc008cgr.2 ; uc008cgs.2 | **TNF_13** | CAAAATTCGAGTGACAAGCCTGTAG | GCTGCTCCTCCACTTGGT | CACGTCGTAGCAAACC |
| uc012arb.2 | **TNF_2** | GCCTCCCTCTCATCAGTTCTATG | CCAGCTGCTCCTCCACTT | CACACTCACAAACCACC |
| uc008cgs.2 | **TNF_3** | CACGCTCTTCTGTCTACTGAACTTC | TCTGGGCCATAGAACTGATGAGA | CCATTTGGGAACTCATCC |
