# Supplementary material for: Changes in the expression of splicing factor transcripts and variations in alternative splicing are associated with lifespan in mice and humans
Source: Aging Cell. 2016 Jun 30;15(5):903–13. doi: 10.1111/acel.12499 (PMC5013025; doi:10.1111/acel.12499)
Supplement: Supplementary file 16 — Data S3 Alternatively expressed isoforms captured by quantitative real‐time PCR assays. Microsoft word file. Additional information describing precisely which alternatively expressed isoforms of analysed genes are captured by each probe set. [file ACEL-15-903-s016.docx]

**Additional Information file 3**: Alternatively-expressed isoforms captured by quantitative real-time PCR assays. Transcript Identifiers refer to Santa Cruz Genome Browser accession numbers (http://www.genome.ucsc.edu/).

**ATM**

Atm-1,3

uc009pme.2

uc009pmd.2

Atm-2

uc012gtj.1

**CDKN2A**

Cdkn2a-1

uc008toi.1

Cdkn2a-2

uc008toh.1

**CHEK2**

Chek2-1

uc008yrw.1

Chek2-2

uc008yrx.1

**FN1**

Fn1-1

uc007bju.2

Fn1-2,5

uc007bjv.2

uc007bjy.2

**LMNA**

Lmna-1

uc008pvj.3

Lmna-1,3

uc008pvj.3

uc008pvl.3

**MYC**

Myc-1

uc007vyh.2

Myc-1,2,3

uc007vyh.2

uc007vyg.2

uc007vyi.1

**TRP53**

Trp53-1,3,4

uc007jql.2

uc007jqm.2

uc007jqn.2

Trp53-2

uc011xww.1

Trp53-3

uc007jqm.2

**VCAN**

Vcan-1

uc007rjg.1

Vcan-2

uc011zck.1

**IL1B**

Il1b-2

uc008mht.1

Il1b-2,3

uc008mht.1

uc008mhu.1

**IL6**

IL6-1,2

uc008wuu.1

uc008wuv.1

Il6-1,3

uc008wuu.1

uc008wuw.1

**NFKB1**

Nfkb1-1,4,5

uc008rly.1

uc012cyg.1

uc008rlx.1

Nfkb1-1,5

uc008rly.1

uc008rlx.1

Nfkb1-2

uc012cyf.1

Nfkb1-3,4,5

uc008rlw.1

uc012cyg.1

uc008rlx.1

Nfkb1-4,5

uc012cyg.1

uc008rlx.1

**STAT1**

Stat1-1

uc007axy.1

Stat1-3,4

uc007axz.1

uc007aya.2

Stat1-2,3,4,5

uc007ayd.2

uc007axz.1

uc007aya.2

uc007ayb.2

Stat1-2,4,5,6

uc007ayd.2

uc007aya.2

uc007ayb.2

uc007ayc.2

Stat1-5

uc007ayb.2

Stat1-6

uc007ayc.2

**TNF**

Tnf-1,2

uc008cgr.2

uc012arb.2

Tnf-1,3

uc008cgr.2

uc008cgs.2

Tnf-3

uc008cgs.2
